# Genome-wide characterization of R2R3-MYB gene family in *Santalum album* and their expression analysis under cold stress

**DOI:** 10.3389/fpls.2023.1142562

**Published:** 2023-03-02

**Authors:** Minqiang Tang, Le Liu, Xu Hu, Haoyue Zheng, Zukai Wang, Yi Liu, Qing Zhu, Licao Cui, Shangqian Xie

**Affiliations:** ^1^ Key Laboratory of Genetics and Germplasm Innovation of Tropical Special Forest Trees and Ornamental Plants (Ministry of Education), School of Forestry, Hainan University, Haikou, China; ^2^ College of Bioscience and Engineering, Jiangxi Agricultural University, Nanchang, China

**Keywords:** R2R3-MYB gene family, cold stress, sandalwood, WGCNA, transcriptome analysis

## Abstract

Sandalwood (*Santalum album*) is a high-value multifunctional tree species that is rich in aromatic substances and is used in medicine and global cosmetics. Due to the scarcity of land resources in tropical and subtropical regions, land in temperate regions is a potential resource for the development of S. album plantations in order to meet the needs of *S. album* production and medicine. The R2R3-MYB transcription factor family is one of the largest in plants and plays an important role in the response to various abiotic stresses. However, the R2R3-MYB gene family of *S. album* has not been studied. In this study, 144 R2R3-MYB genes were successfully identified in the assembly genome sequence, and their characteristics and expression patterns were investigated under various durations of low temperature stress. According to the findings, 31 of the 114 R2R3-MYB genes showed significant differences in expression after cold treatment. Combining transcriptome and weighted gene co-expression network analysis (WGCNA) revealed three key candidate genes (*SaMYB098*, *SaMYB015*, and *SaMYB068*) to be significantly involved in the regulation of cold resistance in *S. album*. The structural characteristics, evolution, and expression pattern of the R2R3-MYB gene in *S. album* were systematically examined at the whole genome level for the first time in this study. It will provide important information for future research into the function of the R2R3-MYB genes and the mechanism of cold stress response in *S. album*.

## 1 Introduction

*Santalum album* L., an important plant belonging to the Class Magnoliopsida, is well known for its medicinal value and valuable carving wood. Unfortunately, owing to its high commercial value and applications in cosmetics, religion, and medicine, the population of *S. album* is gradually declining from overharvesting and illegal trading. This worrisome decline in its population underscores the need for effective *in situ* and *ex situ* conservation strategies. However, owing to the influence of temperature and climate, the natural distribution of sandalwood ranges from 30° north latitude to 40° south latitude, starting from Indonesia in the east, reaching Juan Fernandez Islands in the west, Hawaii Islands in the north, and New Zealand in the south (Kumar et al., 2012). Low temperature is one of the main abiotic factors that hinder the growth and geographical distribution of *S. album.* However, there are very few studies on the mechanisms of environmental adaptation of *S. album*, especially on its mechanisms of adaptation to low temperatures. Therefore, it is of great significance to explore the molecular genetic mechanisms of cold stress for the protection and utilization of *S. album*.

The effects of low-temperature stress on plants are mainly reflected in enzyme activity, membrane system, and cell dehydration that lead to disorders in cell metabolism and even cell death. The response of plants to cold stress at the molecular level is primarily mediated through changes in gene expression, protein levels, and metabolites (Li et al., 2018). The molecular mechanism of cold tolerance has been studied in the model plant, *Arabidopsis thaliana*, and other crop plants, such as maize, rice, wheat, tomato, and barley (Sanghera et al., 2011; Jeon and Kim, 2013). The MYB transcription factors (TFs), which are related to c-Myb, are involved in plant growth and development, metabolism, responses to biotic and abiotic stress, and other biological processes through interaction with the basic helix-loop-helix (bHLH) TFs (Stracke et al., 2001). The MYB TF MdMYB308L in apple was found to positively regulate cold tolerance and anthocyanin accumulation by interacting with MdbHLH33 and enhancing its binding to the promoters of MdCBF2 and MdDFR (An et al., 2020). In *A. thaliana*, the overexpression of the *Mallus baccata* MYB4 (MbMYB4) gene enhanced the tolerance of the transgenic plants to cold and drought stress and cold resistance-related traits, such as the proline and chlorophyll content, and the activity of peroxidase (POD) and catalase (CAT) increased significantly in the transgenic plants (Yao et al., 2022a). Many studies have shown that the MYB gene family is significantly associated with cold stress and cold resistance (Wu et al., 2021; Dar et al., 2022; Yao et al., 2022b). In *A. thaliana*, AtMYB15 negatively regulates the expression of the c-repeat binding factor (CBF) gene, leading to reduced cold resistance (Agarwal et al., 2006). Although considerable progress has been made in research on cold stress in *S. album*, the research has mainly focused on the physiological and phenotypic responses, and studies on the molecular mechanisms are limited (Zhang et al., 2017; Yan et al., 2019). Specifically, whole-genome identification of the MYB gene family and its expression pattern under cold stress has not yet been reported in *S. album*.

The MYB gene family plays an important role in plant evolution. Generally, a gene family consists of a group of genes from a common ancestor whose members share > 50% pairwise amino acid sequence similarity and contain a common functional domain (Thornton and DeSalle, 2000). The MYB gene family constitutes one of the largest TF families in plants. The members of this family contain a unique conserved MYB domain, which is composed of 1–4 tandem and non-repetitive R motifs. Based on the number of MYB domains, the MYB genes can be classified into four categories: 1R-MYB/MYB-related (1R motif), R2R3-MYB (2R motifs), 3R-MYB (3R motifs), and 4R-MYB (4R motifs) (Dubos et al., 2010). R2R3-MYB genes are the most abundant subtype in plants; they contain two R structures (R2R3) at the N-terminal and generally contain a transcriptional activation domain at the C-terminal. They are involved in cell differentiation, hormone response, secondary metabolism, and environmental stress. Yao et al. performed genome-wide analysis of the R2R3-MYB gene family members in ginger (*Zingiber officinale* Roscoe) and identified 120 segmental duplications in R2R3-MYB using gene duplication analysis. Ten of the R2R3-MYB genes were significantly differentially expressed under abscisic acid (ABA) and low-temperature stress in ginger leaves (Yao et al., 2022). Zhang et al. identified 69 R2R3-MYB proteins containing a conserved functional domain in *Cyclocarya paliurus*, four of which were found to respond to salt stress by regulating plant hormone signals (Zhang et al., 2022). Yuan et al. (2021) identified 202 R2R3-MYB genes in the polyploid *Saccharum spontaneum* genome sequence. They used collinearity analysis and found that 70% of the genes had experienced duplication events, suggesting the contributors to the MYB gene family expansion. Four of these R2R3-MYB genes actively responded to drought treatment in stress expression analysis (Yuan et al., 2021). However, genome-wide identification of 2R3R-MYB genes and their responses to stress have not yet been reported in *S. album*.

Weighted gene co-expression network analysis (WGCNA) is a data mining method used to analyze the gene expression patterns in multiple samples, which can be used to analyzing the correlation between genes, identifying modules with high phenotypic correlation, and identifying hub genes in different modules (Langfelder et al., 2008). Sharma et al. performed meta-analysis and WGCNA on 390 samples from 29 studies in *A. thaliana* and identified 6,120 and 7,079 differentially expressed genes (DEGs) under drought and cold stress, respectively. They also found that 28% of the DEGs were common to both drought and cold stress, and most of them showed a similar expression pattern (Sharma et al., 2018). Using WGCNA at the transcriptome level, Zeng et al. provided a potential regulatory mechanism for cold stress and recovery of rice cultivars and identified key candidate genes involved in cold tolerance, which provided valuable information for cultivating rice strains with high cold tolerance in the future (Zeng et al., 2022). Li et al. used time-lag initiation of the two pathways and WGCNA in *Arabidopsis* to demonstrate that vernalization was independent of cold acclimation. WGCNA revealed three main networks involving response of ethylene and jasmonic acid, chromatin modification, and cold adaptation in response to prolonged cold exposure, which provided a comprehensive overview of the global changes mediated by cold stress and vernalization in *Arabidopsis* (Li et al., 2021). In the National Center for Biotechnology Information (NCBI) database, transcriptome datasets for 41 samples involving different tissues and abiotic stress were found for *S. album*. However, because the quality of the reference genome was not optimal, these datasets have not been used effectively at present, especially for WGCNA analysis and identification of related functional genes. Re-mining these important data will help to identify the molecular genetic basis of cold tolerance in *S. album*.

In this study, we performed whole-genome identification and characterization of the R2R3-MYB gene family in *S. album* and revealed the classification and evolution of 75 plants. Furthermore, we performed transcriptome analysis and WGCNA of the RNA-seq data under cold stress at four time points and identified key R2R3-MYB genes responsive to cold stress. Our study provides a genome-wide overview, identifies the expression pattern of R2R3-MYB genes under cold treatment, and reveals the identity of key genes for improving cold resistance and *ex situ* protection of *S. album*.

## 2 Materials and methods

### 2.1 Data sources

We obtained a chromosome-level reference genome sequence of *S. album*. The reference genome size was 236.49 million bases (Mb) and included 28,665 coded proteins. For genes with multiple transcripts, the longest transcript was selected for subsequent analysis. The transcriptome and phenotypic data following cold treatments for 0, 12, 24, and 48h were obtained from a published article (Zhang et al., 2017). A total of 12 RNA-seq datasets were collected from the NCBI database under BioProject accession number PRJNA320980, which comprised three replicates of four time points of 0, 12, 24, and 48h at 4°C. The consistency of three replicability at the same time point was acceptable as shown in the previous research article (Zhang et al., 2017).

### 2.2 Identification of R2R3-MYB genes in *S. album*

Genome sequence and gene annotation information for *S. album* were acquired by our research group (unpublished data). The hidden Markov model (HMM) of the MYB domain was downloaded from the Pfam database (PF00249), and the *S. album* MYB genes were searched in the protein database *via* an HMM search using HMM files (*E* < 1 × 10^−5^) (Finn et al., 2011). Candidate MYB protein sequences, CDS, and conserved domain sequences were extracted using a Perl script (**Supplementary File S1**). The candidate protein sequences were aligned with Pfam (http://pfam.xfam.org/), SMART (http://smart.embl.de/smart/batch.pl), and CDD (https://www.ncbi.nlm.nih.gov/cdd/) databases to predict the conserved domains. There is a conserved DNA binding domain (DBD) in MYB proteins, made up of 1–4 imperfect amino acid sequence repeats “R.” There are approximately 52 amino acids in each “R”, forming every similar folding architecture with three well-defined α-helixes. The helix-turn-helix hydrophobic core was formed by the second and third helices of each “R” with three regularly spaced tryptophans (W) or other hydrophobic residues. MYB superfamily is divided into four subfamilies based on the number of “R” in MYB DBD: MYB-related subfamily gene with a single or partial “R”, R2R3-MYB subfamily gene with “R2” and “R3”, 3R-MYB subfamily gene with “R1”, “R2”, and “R3” as well as 4R-MYB subfamily gene with four “R1/R2” A total of 154 MYB genes were ultimately identified in *S. album*, of which 31 were clustered into 1R-MYB type, 114 into R2R3-MYB type, two into 3R-MYB type, and the remaining seven genes could not be clustered in any group with certainty. The online tool ExPASy (https://web.expasy.org/protparam/) was used to analyze the amino acid number, isoelectric point (pI), and molecular weight of the R2R3-MYB proteins. Plant-mPLoc (http://www.csbio.sjtu.edu.cn/bioinf/plant-multi/) predictor was used to predict the subcellular localization of the R2R3-MYB proteins.

### 2.3 Phylogenetic analysis, classification of R2R3-MYB genes, and gene duplication analyses

Multiple alignments of R2R3-MYB amino acids were conducted using ClustalW with the default parameters. A phylogenetic tree containing MYB proteins of *S. album* and 75 other plants was constructed using the maximum likelihood (ML) method in MEGA7.0 (Kumar et al., 2016). The parameters were as follows: Poisson model, pairwise deletion, and 1,000 bootstrap replications (Edgar, 2004). The R2R3-MYB family of genes from 75 plants was used as a reference for the classification of R2R3-MYB family members in *S. album*.

Conserved motifs in the R2R3-MYB proteins from *S. album* were analyzed using the online tool MEME (http://meme-suite.org/) with the following parameters: maximum number of motifs = 20 and optimum width = 6–100 residues (Bailey et al., 2009). Finally, the conserved motifs and domains were visualized using the TBtools software (Chen et al., 2020). The distribution of 114 R2R3-MYB genes identified in the reference genome annotation information of *S. album* was mapped to chromosomes and visualized using the software MG2C (Chao et al., 2021). MCScanX is used to analyze tandem and segmental gene duplications (Wang et al., 2012). All R2R3-MYB protein sequences of *S. album* were compared against themselves using BLASTP, with tabular output format (-m 8) and an e value of < 1e −10. The BLASTP tabular file and a simplified *S. album* gene location file served as inputs for MCScanX to identify duplication types using default settings (Tang et al., 2014). Values of nonsynonymous (Ka) and synonymous (Ks) substitution rates were calculated using KaKs_Calculator software (Zhang et al., 2006).

### 2.4 Expression pattern of R2R3-MYB genes

All RNA sequence datasets from *S. album* leaves at the four cold treatment time points were filtered using Fastp with default parameters (Chen et al., 2018). The total filtered high-quality clean RNA-seq data were mapped to the reference genome of *S. album* (not officially published) using Hisat2 with default parameters (Kim et al., 2019). The mapped reads with mapping quality (MQ) ≤ 30 were filtered using Samtools, and BAM files were sorted. StringTie (Pertea et al., 2015) was used to count unique and normalized mapped reads as fragments per kilobase per million (FPKM) mapped reads for each gene with the parameter of “-B -e -G.” Transcriptome gene expression data were visualized using ImageGP software (Chen et al., 2022). The R2R3-MYB gene expression data are listed in **Supplementary Table S2**.

### 2.5 WGCNA analysis and prediction of the three-dimensional structure of key genes

To identify the core gene modules and the hub genes within the modules related to cold stress, we performed a WGCNA (Langfelder and Horvath, 2008). We evaluated 10,483 genes after filtering total FPKM ≤ 100 at the four time points and three biological replicates to test their availability and used the R package termed “WGCNA” to construct a gene co-expression network. Candidate power values were set from 1 to 30. The threshold for scale-free topology model fit was set at 0.8. Subsequently, we constructed an adjacency matrix to describe the correlation strength between the modules and cold resistance-related traits, which were malondialdehyde (MDA), superoxide dismutase (SOD), peroxidase (POD) activity, soluble sugar content, intercellular CO2 concentration, and respiration rate. These phenotypic data were collected from the previous research article (Zhang et al., 2017). Correlations between modules and cold stress-related traits were calculated using a function in “moduleTraitCor.” Finally, the genes in the modules with high correlation with cold stress-related traits were extracted and compared with the significant DEGs under different cold treatments. Genes present in both DEGs and modules were considered as the key genes associated with cold stress in *S. album*.

The spatial structure of genes determines their functions. The functions and three-dimensional (3D) structures of the key genes were predicted using the Swiss-Model website. From the database, sequences with coverage greater than 50% were selected as templates for structural prediction. The qualitative model energy analysis (QMEAN) values of > 0.6 for predicted structures was considered reliable.

## 3 Results

### 3.1 Identification and analysis of physicochemical properties of R2R3-MYB genes

Genome sequence and gene annotation information for *S. album* were obtained by our research group (unpublished). To determine the conserved sequence of the MYB protein in *S. album*, the conserved domains of the MYB genes in *S. album* were identified through alignment based on the classical HMM (PF00249). Two comparison searches were performed and online sites such as Simple Modular Architecture Research Tool (SMART), Pfam, and National Center for Biotechnology Information-Conserved Domains Database (NCBI-CDD) were used to verify the structures of the conserved domains in the candidate genes. The remaining seven MYB genes could not be classified with certainty. A total of 114 R2R3-MYB genes were named based on their subfamilies as SaMYB001–SaMYB114 (**Supplementary Table S1**; **Supplementary File S2**). These genes were distributed across 10 chromosomes. The physicochemical properties of the MYB proteins were analyzed online using the ExPaSy tool. The results showed that the 114 MYB proteins contained 169–1452 amino acids, with a molecular weight of 18.36–159.44 kDa. SaMYB087 contained the lowest number of amino acids (169 aa) and consequently had the least molecular weight (18.36 kDa). The mean theoretical pI of all the R2R3-MYB genes was 7.03 (range: 4.71–9.58). The theoretical pI of 64 R2R3-MYB proteins was < 7, indicating that these proteins were acidic, whereas that of the remaining 50 R2R3-MYB proteins was > 7, indicating that these were alkaline. The predicted subcellular localization of all the proteins was nuclear, and only SaMYB049 was distributed in the chloroplast and nucleus (**Supplementary Table S1**).

### 3.2 Phylogenetic analysis and characterization of R2R3-MYB genes in S. album

**Figure 1:**
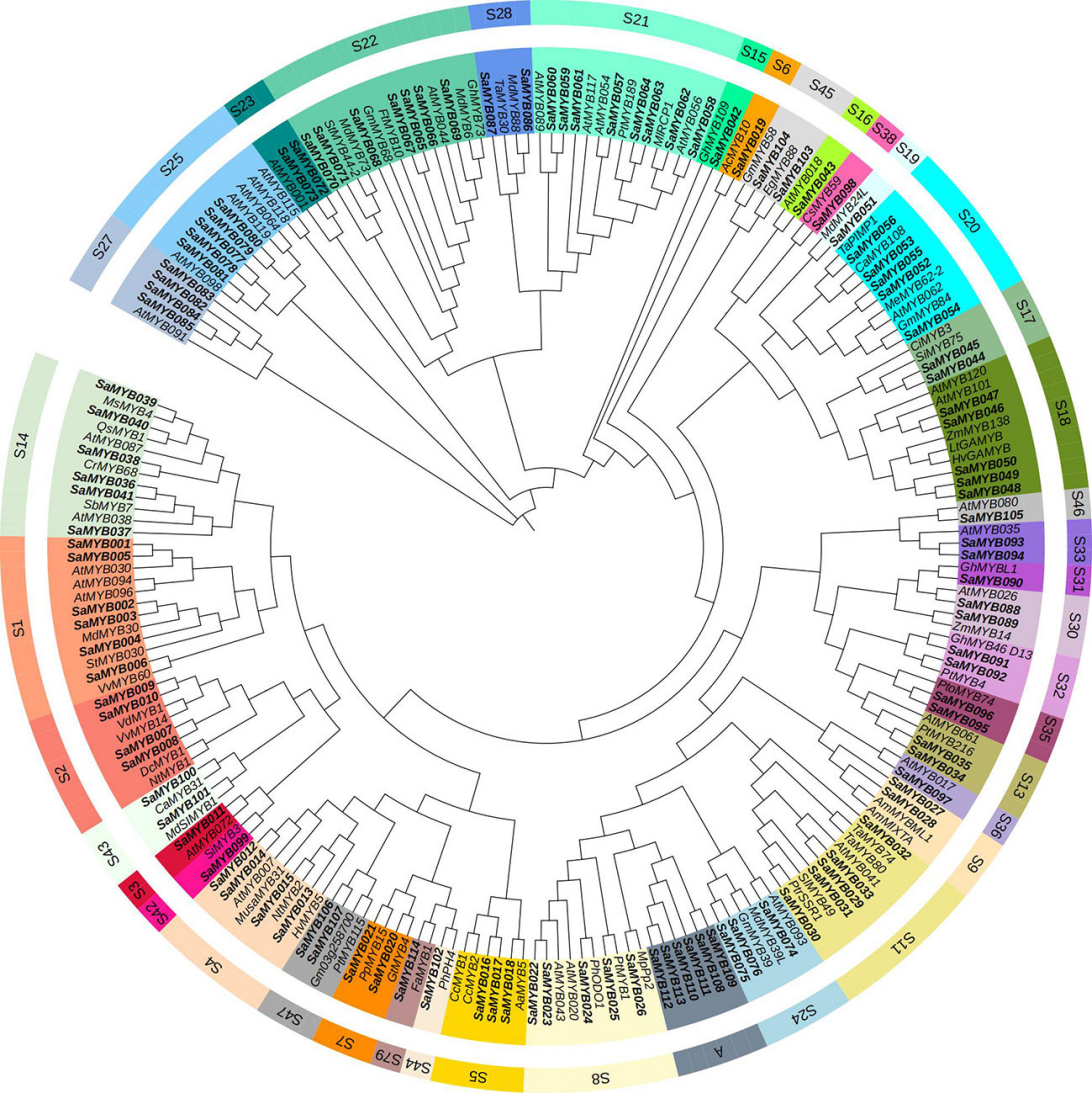
Result of phylogenetic analysis of R2R3-MYB proteins in *S. album*. Same color font of genes represent that they were in common subfamilies.

The R2R3-MYB protein sequences of *S. album* and 75 other plants (Wu et al., 2022) were aligned using ClustalW program and a phylogenetic evolution tree was constructed. Toward subfamily classification of the R2R3-MYB proteins (Wu et al., 2022), the 114 MYB proteins in *S. album* were divided into 40 categories (**Figure 1**; **Supplementary Table S1**): the S21 category in watchet, with the largest number of eight R2R3-YMB genes in *S. album* and the S22 category in cyan, with the second largest number of seven R2R3-MYB genes in *S. album*. The number of SaR2R3-MYB genes in each subgroup was close to that of R2R3-MYB genes in *A. thaliana*. Interestingly, all genes in subfamily A were R2R3-MYB genes in *S. album*, and no genes from any other species were clustered with them, which indicates that Subfamily A may be unique to *S. album*. Genes of the same subfamily exhibit high homology and sequence similarity and may have similar gene functions. The S21 subfamily harboring eight R2R3-MYB genes in *S. album* is related to specialized metabolic processes, such as cell wall thickening, seed oil accumulation, and phenylpropanoid metabolism. The S22 subfamily, harboring seven R2R3-MYB genes in *S. album*, is related to abiotic stress. The genes in the S1 and S2 subfamilies were related to abiotic stress, such as salt tolerance, drought stress, and cold tolerance.

**Figure 2:**
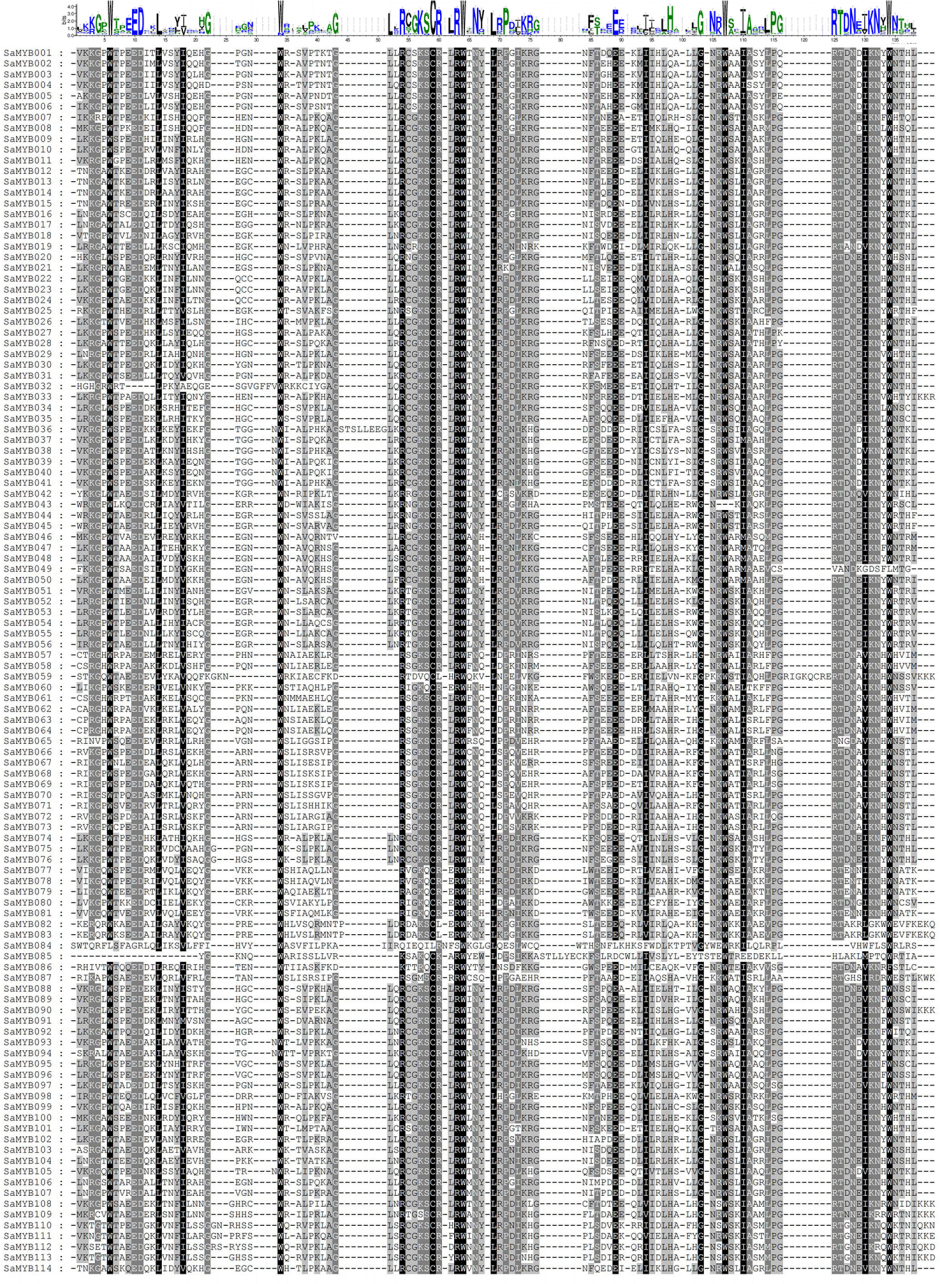
Results of conserved motifs and sequence alignment analyses of 114 R2R3-MYB proteins in *S.album*.

**Figure 3:**
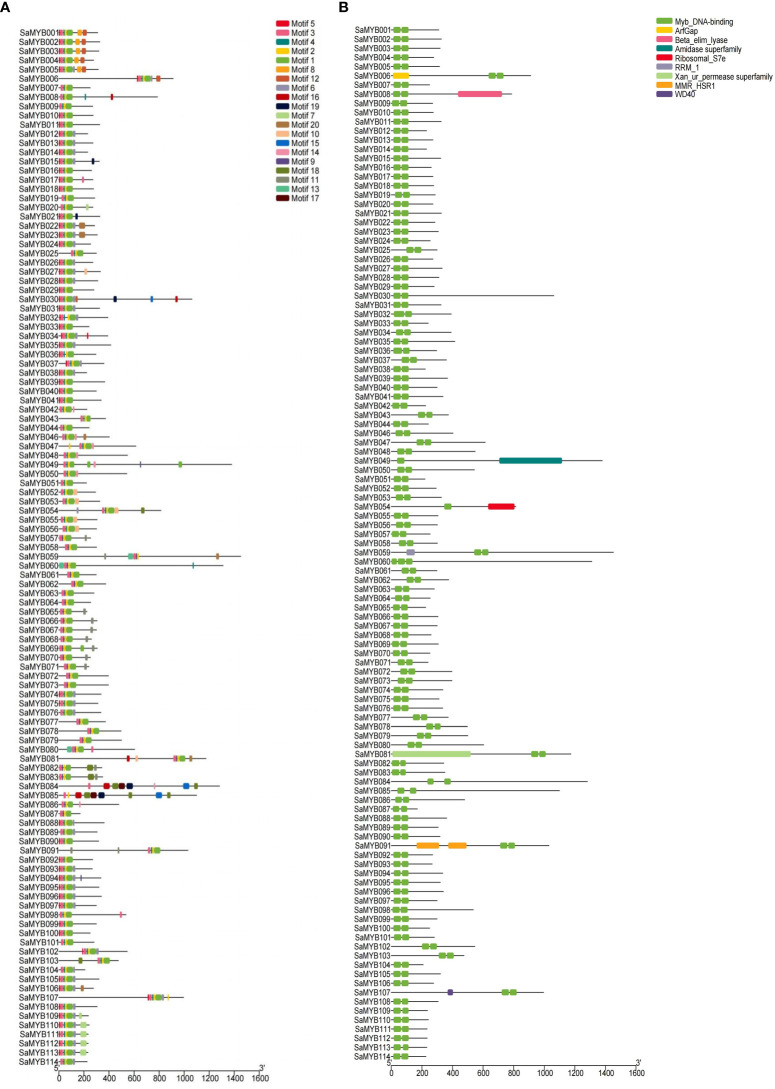
Conserved motif analyses of the 114 R2R3-MYB genes in *S. album*. **(A)** The left panel shows the results of conserved motif analysis. A total of 20 predicted motifs are represented by different colored boxes. **(B)** The right panel shows the conserved domains in the 114 R2R3-MYB genes with different colors representing the different types of domains.

MEGA7 and WEBLOGO softwares were used to conduct multiple sequence alignments and to characterize the conserved regions in the 114 R2R3-MYB proteins in *S. album*. **Figure 2** shows that the R2 structure contains three very conserved tryptophan (W) residues, and every two W residues are separated by 19 amino acids. The R3 structure contains two very conserved W residues: The first W is replaced by phenylalanine (F), isoleucine (I), and leucine (L) and the second and third W residues are separated by 18 amino acids. The online software MEME was used to analyze the conserved domains in R2R3-MYB proteins (**Figure 3**). Analysis of the conserved motifs revealed that all R2R3-MYB proteins harbored motif 1 (green box), motif 2 (yellow box), and motif 3 (pink box), as shown in **Figure 3A**. All R2R3-MYB proteins in *S. album* contained conserved MYB DNA-binding domains (**Figure 3B**). These conserved amino acids and structures indicate that the R2R3-MYB genes identified in our study are reliable.

### 3.3 Gene duplication and chromosomal location of R2R3-MYBs in *S. album*

**Figure 4:**
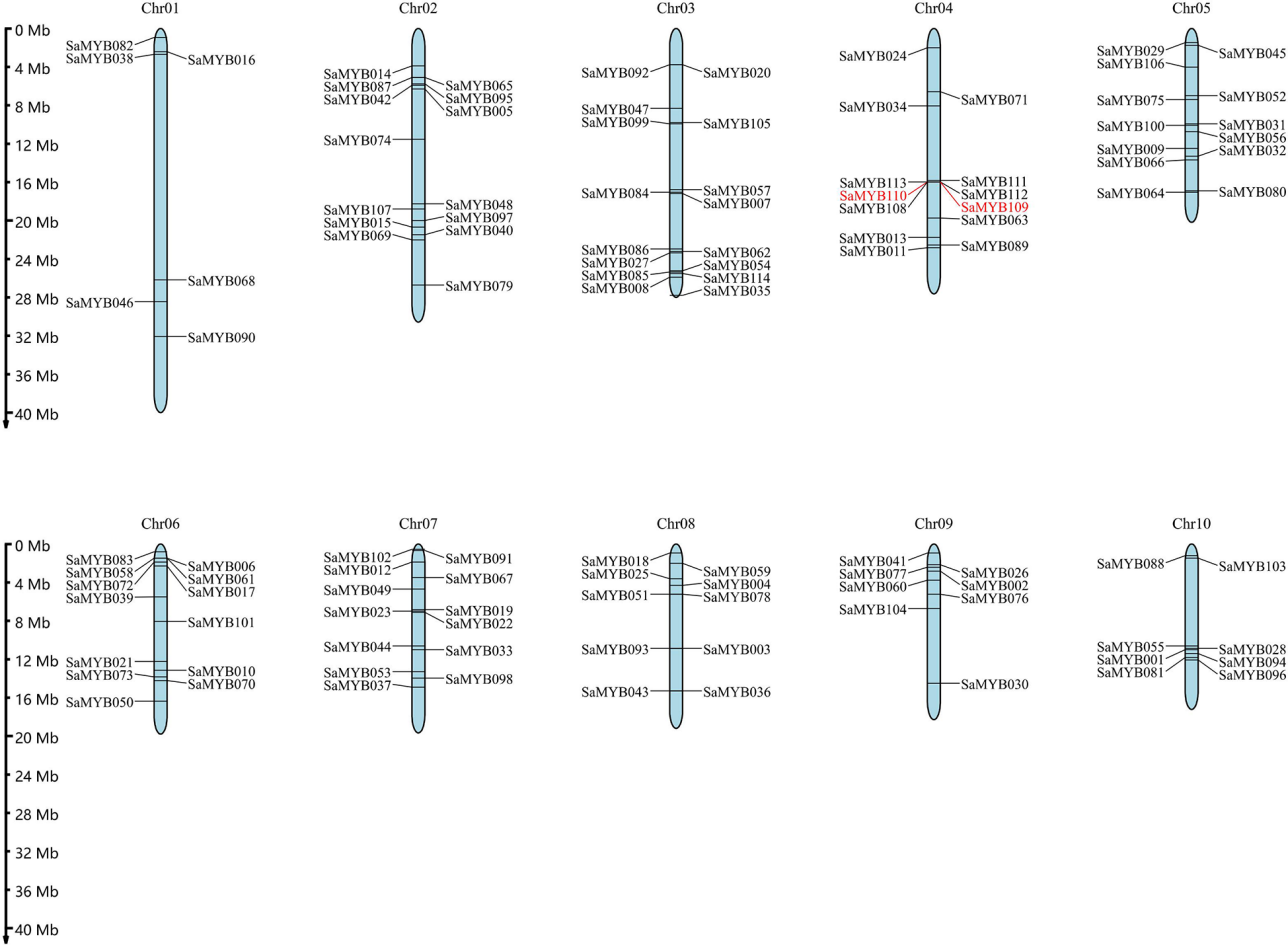
Chromosomal locations of the 114 R2R3-MYB genes in *S. album*. The ruler on the left indicates the physical position of reference genome. The pair of tandem duplicated genes is shown in red font.

The location of the R2R3-MYB genes was extracted from the gene annotation general feature format (gff) file of *S. album*. The results indicated that the 114 R2R3-MYB genes were unevenly distributed on ten chromosomes (**Figure 4**): six genes in chromosome 01 (5.26% of the total); 14 genes in chromosome 02 (12.30%); 16 genes in chromosome 03 (14.04%) with the most genes; 13 genes each in chromosome 04, chromosome 05, chromosome 06, and chromosome 07 (11.40%); 10 genes in chromosome 08 (8.77%); and eight genes each in chromosome 09 and chromosome 10 (7.02%).

On one of the aforementioned ten chromosomes was a pair of tandem duplicate genes, specifically distributed in chromosome 04 (SaMYB109 and SaMYB110). The two genes belong to Subfamily A, and the distance between them was 8116 bp. Their nonsynonymous rate (Ka) and synonymous rate (Ks) substitution rates were analyzed, the non-synonymous mutation rate was 0.39, and the synonymous mutation rate was 1.75. The Ka/Ks ratio was found to be 0.22 (**Table 1**), which was less than 1, indicating negative selection pressure. In addition to tandem duplication, MCScanX was used to analyze fragment duplication events in the R2R3-MYB gene family. The results showed that 33.33% (38/114) of the R2R3-MYB members showed segmental duplication. The Ka/Ks values of SaMYB103 and SaMYB017 were > 1 (1.17), implying that they evolved under positive selection pressure. The Ka/Ks value of the remaining gene pairs was < 1, indicating that they evolved under the effect of purifying selection (**Table 1**). Thus, the analysis of duplication events in the R2R3-MYB genes suggests that some genes were produced by tandem and segmental duplication, and these gene duplication events may be among the driving forces of gene evolution.

**Table 1:** Ka, Ks and Ka/Ks of replication pairs of R2R3-MYB gene family in *S. album*.

Duplicated gene pairs	Non synonymous (Ka)	Synonymous (Ks)	Ka/Ks	Duplicated type
SaMYB110 & SaMYB109	0.39	1.75	0.22	tandem
SaMYB003 & SaMYB002	0.17	0.85	0.19	segmental
SaMYB004 & SaMYB003	0.17	0.84	0.20	segmental
SaMYB005 & SaMYB001	0.25	1.19	0.21	segmental
SaMYB009 & SaMYB010	0.20	0.70	0.28	segmental
SaMYB012 & SaMYB014	0.07	0.66	0.11	segmental
SaMYB016 & SaMYB017	0.28	1.16	0.24	segmental
SaMYB029 & SaMYB033	0.24	1.21	0.20	segmental
SaMYB036 & SaMYB041	0.21	0.93	0.22	segmental
SaMYB044 & SaMYB045	0.12	1.04	0.11	segmental
SaMYB059 & SaMYB060	0.21	0.50	0.43	segmental
SaMYB063 & SaMYB064	0.19	0.92	0.21	segmental
SaMYB065 & SaMYB067	0.35	0.74	0.48	segmental
SaMYB066 & SaMYB067	0.36	1.79	0.20	segmental
SaMYB068 & SaMYB069	0.15	0.68	0.23	segmental
SaMYB072 & SaMYB073	0.32	1.79	0.18	segmental
SaMYB077 & SaMYB078	0.26	0.94	0.28	segmental
SaMYB081 & SaMYB078	2.08	NA	NA	segmental
SaMYB082 & SaMYB083	0.07	0.57	0.13	segmental
SaMYB084 & SaMYB085	1.61	1.94	0.83	segmental
SaMYB095 & SaMYB096	0.18	1.04	0.18	segmental
SaMYB103 & SaMYB017	2.07	1.77	1.17	segmental

### 3.4 SaMYB gene expression pattern under cold stress

**Figure 5:**
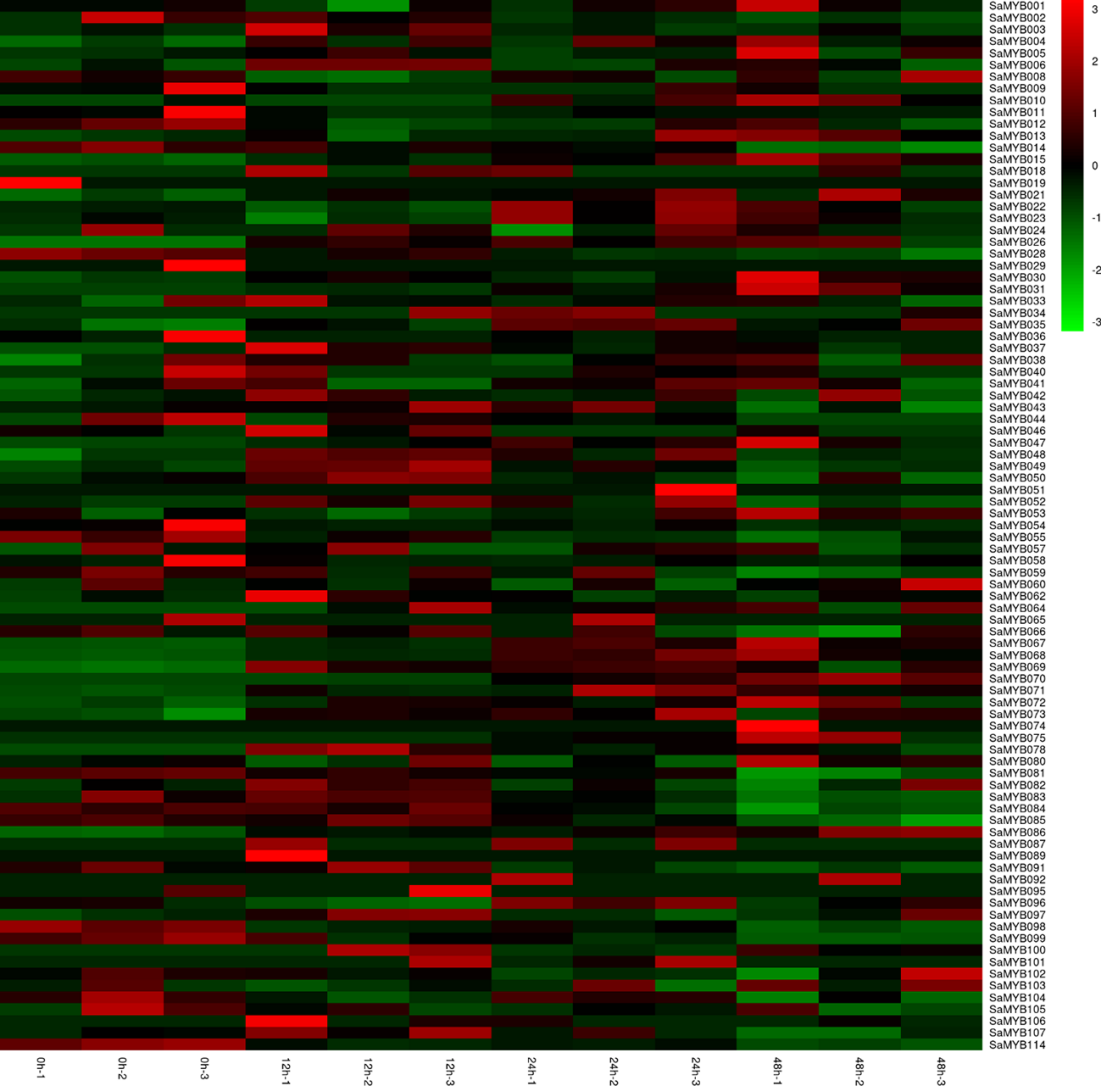
R2R3-MYB gene expression levels determined by RNA-seq at the four time points of cold (4°C) treatment. Expression profiles were normalized to log_10_(FPKM). The color scale represents relative expression level from low (green) to high (red) values.

We collected transcriptome datasets corresponding to 6-month-old *S. album* seedling leaves subjected to 4°C treatment for 0 (0h), 12 (12h), 24 (24h), and 48 (48h) using three biological replicates from the NCBI database generated in a previous study (Zhang et al., 2017). After quality control assessments, a total of 7.62 Gb clean data was retained, Q30 of which accounted for over 91.25%. We used the Hisat2 alignment program to map the qualified RNA-sequencing data on our reference genome (unofficially published). The mapping rate ranged from 93.85 to 95.81%, with an average of 95.11%, which indicated that these RNA-sequence data were reliable to quantify the global abundance of R2R3-MYB gene expression following cold treatment. The square of the Pearson’s correlation coefficient (r) was > 0.91 among the three biological replicates at each time point, indicating both operational stability and reliability of the experimental results. Of the 114 R2R3-MYB genes, 89 were found to be expressed at least once time. The expression patterns of the R2R3-MYB genes in *S. album* under different cold-stress conditions were visualized using heatmap analysis. The results showed that the expression levels of 31 R2R3-MYB genes were significantly up-regulated (log_2_[fold change] > 1) at least one time point of cold stress (**Figure 5**; **Supplementary Table S2**), whereas that of 13 R2R3-MYB genes were significantly down-regulated at least one time point of cold treatment. SaMYB078 and SaMYB100 were not expressed at 0h at 4°C. However, they were expressed at 12, 24, and 48h after cold treatment, which indicated that the two genes may be involved in cold-stress resistance. Five genes (SaMYB031, SaMYB068, SaMYB069, SaMYB004, and SaMYB015) were highly expressed following cold treatment at three different time points, whereas SaMYB114 and SaMYB058 were down-regulated at three time points of cold treatment (**Figure 5**; **Supplementary Table S2**).

### 3.5 Identification of key R2R3-MYB genes related to cold stress based on WGCNA

**Figure 6:**
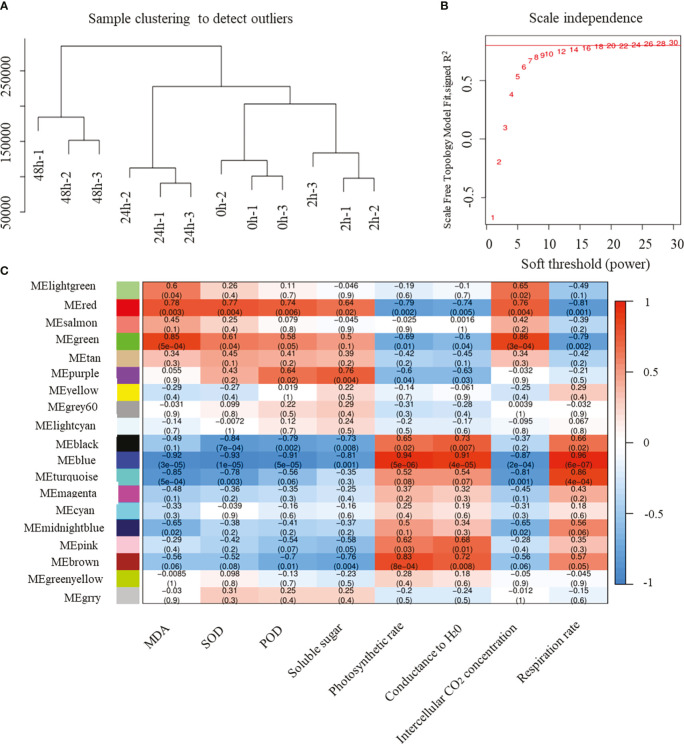
Results of WGCNA based on the gene expression level and phenotypic data. **(A)** Results of sample clustering; **(B)** results of scale independence, candidate power was from 1 to 30; **(C)** relationship of different modules and cold resistance-related traits. Numbers in the box represent the correlation coefficient, and the number in the brackets represent the corresponding *p*-value. The color scale represents correlation coefficient from 1 (red) to -1 (blue).

WGCNA can be used to identify modules of highly correlated genes, to summarize such clusters using the module eigengene or an intramodular hub gene, and to relate modules to one another and to external sample traits. Here, we used WGCNA to identify the hub R2R3-MYB genes associated with cold stress. After removing genes with a total FPKM ≤ 100 at four time points and three biological replicates, a total of 10,483 genes were reserved for WGCNA. Correlation coefficient cluster analysis of the expression levels of 12 samples revealed that the clustering among samples was acceptable and there were no outliers (**Figure 6A**). Twenty candidate power values from 1 to 30 were set for model fitting. The results showed that, when the power value was 20, there was an obvious inflection point where the topology model fit value was 0.8 and stable (**Figure 6B**). Therefore, we chose a power value of 20 for module construction and clustering. Based on correlation analysis and clustering according to the FPKM value of different genes, the genes with high correlation were allocated to the same module. Different genes were divided into 17 modules according to their co-expression patterns, with different colors representing the different modules (**Figure 6C**; **Supplementary Table S3**). For correlation analysis between the trends in gene expression modules and cold stress-related traits, the Pearson correlation coefficient (*r* > 0.5) and *P* < 0.05 were set as thresholds. The MEred and MEgreen modules were found to significantly positively correlate with changes in malondialdehyde (MDA), superoxide dismutase (SOD), peroxidase (POD) activity, soluble sugar content, and intercellular CO_2_ concentration in response to cold stress, whereas they negatively correlated with photosynthetic rate, conductance to H_2_O, and respiration rate. MEblue was most significantly positively correlated with the photosynthetic rate, conductance to H_2_O, and respiration rate; the correlation coefficients were up to 0.9. The MEblue module was most significantly negatively correlated with MDA, SOD, POD activity, soluble sugar content, and intercellular CO_2_ concentration. This indicated that the genes in these significantly correlated modules were the core genes involved in cold-stress response.

**Figure 7:**
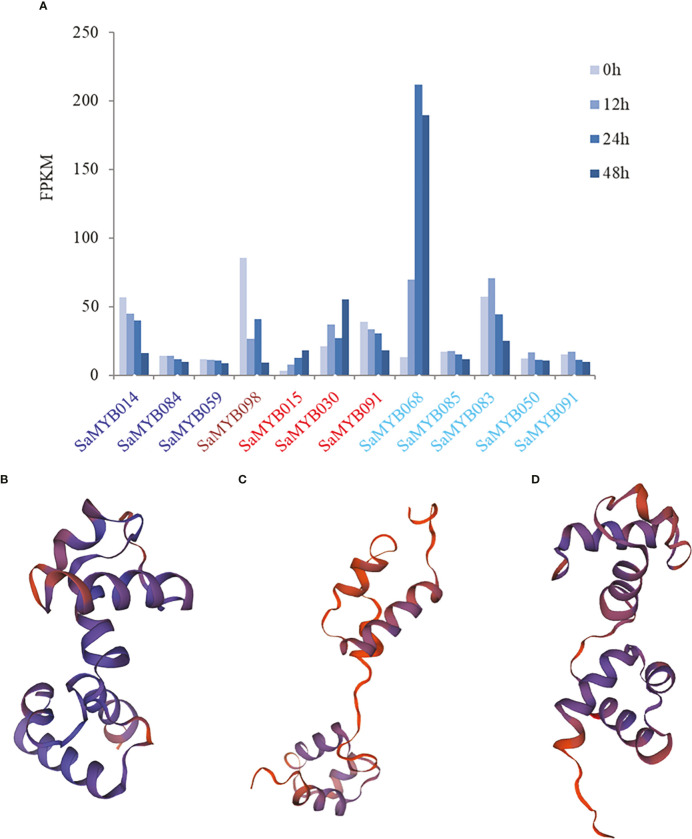
The expression patterns of module genes and the protein structure prediction results of the three important genes. **(A)** color of the text in the *x*-axis represents the corresponding module in same color in **Figure 6**. The *y*-axis represents the gene expression level; **(B)** the prediction result of protein spatial structure of *SaMYB098*; **(C)** the prediction result of protein spatial structure of *SaMYB01*; **(D)** the prediction result of protein spatial structure of *SaMYB068*.

Further analysis revealed that the MEblue module contained three (SaMYB014, SaMYB084, and SaMYB059), MEbrown module contained one (SaMYB098), and MEred module contained three R2R3-MYB genes (SaMYB015, SaMYB030, and SaMYB081). The MEturquoise module contained five R2R3-MYB genes (SaMYB068, SaMYB085, SaMYB083, SaMYB050, and SaMYB091). Combined with the expression levels of these genes at different durations of cold stress, the expression levels of three core genes were significantly different under cold stress treatment. The expression level of SaMYB098 was 85.49 at 0h cold treatment, but decreased to 26.55 at 12h cold treatment, and further decreased to 9.32 after 48h cold stress, indicating that SaMYB098 may be negatively regulated to cold stress. The expression of SaMYB015 in the MEred module was low at 0h (FPKM = 3.18); however, it increased progressively after cold stress treatment, and the expression levels at 12, 24, and 48h were 7.73, 12.87, and 18.18, respectively. The expression level of SaMYB068 in the MEturquoise module was similar to that of SaMYB015 (**Figure 7A**). The 3D structure prediction analyses of the three important genes using the Swiss-Model online tool revealed that, although their structures were different at the 3D level (**Figures 7B–D**, QMEAN Z-scores > 0.65), they all harbored a classical R2R3-MYB binding domain. Therefore, it is suggested that these three genes may participate in cold stress in different ways. Overall, using a combination of gene family identification, transcriptome data, and WGCNA analysis, three core genes with differential expression significantly related to cold stress were identified, which can provide important information for the genetic improvement of *S. album* under cold stress.

## 4 Discussion

Among the many transcription factor families in plants, the MYB family constitutes one of the largest transcription factor families. MYB proteins regulate plant growth and development, primary and secondary metabolism, and response to various abiotic stresses, such as drought, cold, and salt (Dubos et al., 2010; Chen et al., 2019; Millard et al., 2019). Genome-wide identification of the MYB gene family has been performed in various plants, such as Arabidopsis (Matus et al., 2008), *Cucumis sativus* (Li et al., 2012), maize (Dias et al., 2003), and wheat (Gao et al., 2016). The members of the plant MYB gene family contain 1–4 incomplete MYB repeat sequences and can be divided into four subfamilies based on the number of repeat sequences: 1R-MYB type (containing one or two separate repeat sequences), R2R3-MYB type (containing two adjacent repeat sequences), and 3R-MYB type and 4R-MYB type (containing three and four adjacent repeat sequences, respectively). Among these, the R2R3-MYB gene family is most abundant in Plants (Jiang and Rao, 2020; Tombuloglu, 2020). However, the whole-genome MYB gene family has not yet been identified in *S. album*. In this study, the MYB gene family in *S. album* was identified at the whole-genome level. The physical and chemical properties, physical tree, preserved motifs, and gene duplications were systematically analyzed. Consequently, 154 MYB genes in *S. album* were identified, 114 of which were classified as R2R3-MYB genes. The R2R3-MYB gene family in plants contains two R structures at the N-terminal. The number of R2R3 genes in *S. album* was similar to that in other plants, such as 125 in Arabidopsis (Stracke et al., 2001), 119 in pea (Yang et al., 2022), 114 in moso bamboo (Hou et al., 2018), and 133 in white clover (Ma et al., 2022), indicating that the results for the R2R3 gene family in *S. album* were reliable. The functions of R2R3 MYBs can be divided into three main processes: development and cell differentiation, specialized metabolism, and stress response. Du et al. classified the gene family into 73 subfamilies based on their highly conserved domain and motif composition (Du et al., 2015; Wu et al., 2022). Through multiple sequence alignment and evolutionary analysis, the 114 *S. album* MYB proteins were divided into 40 categories (**Figure 1**; **Supplementary Table S1**). Thirty-nine of the 40 subfamilies could be clustered with the corresponding subfamilies of other plants. Only Subfamily A contained six genes (SaMYB108, SaMYB109, SaMYB110 SaMYB111, SaMYB112, and SaMYB113), indicating that these genes may participate in specific biological processes in *S. album*.

Gene duplication occurs through various mechanisms, such as tandem duplication, gene transformation, horizontal transfer and other translocations, hybridization, duplication, and doubling of the whole segment of the chromosome during the recombination process, resulting in effective doubling of the large-scale genome sequence (Sankoff, 2001). Regardless of the origin, duplication of genes in a genome can lead to increased expression of the gene. Differentiation of duplicated genes may be subject to sub-functionalization and produce neo-functionalization to enhance the ability of plants to adapt to the environment and respond to various stresses (Liu and Adams, 2007; Airoldi and Davies, 2012). In this study, a pair of tandem duplicated genes was identified in the R2R3-MYB gene family in *S. album*. About one-third of R2R3-MYB genes have replication events, indicating that these genes may participate in special biological processes in *S. album*. Nonsynonymous (Ka) and synonymous (Ks) substitution rates are important indicators for studying the pressures of gene selection, identifying deviations from neutrality, and estimating the occurrence time of replication events (Hurst, 2002; Echave et al., 2016). A comparative analysis of the positively and negatively selected genes was performed based on Ka and Ks, and artificial selection was found to be a major factor affecting the gene evolutionary rate, compactness, expression level, and genetic diversity in barley (Tao et al., 2022). Song et al. compared negatively and positively selected genes in *Arachis duranensis* and *Arachis ipaënsis* and found Ks to be a determining factor that affected the selection pressure (Song et al., 2018). In 22 pairs of duplicate genes in our study (excluding one pair [SaMYB110 and SaMYB109] with Ka/Ks value > 1), the Ka/Ks value of the duplicated genes was < 1 (range: 0.11–0.83), which indicates that most of the R2R3-MYB genes in *S. album* were subjected to purifying selection.

Although the non-MYB regions diverged in different plant species, the two conserved MYB structures are a signature feature of R2R3-MYB genes and include two main functional parts: a DNA-binding domain at the N-terminal and a regulatory region at the C-terminal (Wu et al., 2022). In this study, 114 R2R3-MYB genes harboring highly conserved R2R3 sequences were identified in *S. album* (**Figures 2**, **3**), and the subcellular localization results showed that all of them localized to the nucleus excepting SaMYB049 distributed in the chloroplast and nucleus. These results are consistent with those reported for other plants (Stracke et al., 2001; Wilkins et al., 2009), indicating that the R2R3-MYB genes identified in this study are reliable. Although the R2R3-MYB genes have highly conserved sequences, they are functionally distinct. Some of them are essential for plant development and cell differentiation (Oshima et al., 2013; Liu et al., 2015), whereas some serve as key regulators of responses to diverse environmental stresses (abiotic and biotic stresses), such as drought, temperature, and salinity (Li et al., 2015). Some of them play an important role in specialized metabolic biosynthesis pathways (Stracke et al., 2001; Dubos et al., 2010). In this study, three key candidate genes that are significantly related to cold stress were identified through genome-wide identification of the R2R3-MYB gene family, transcriptome analysis, and WGCNA. Comparison of the 3D structure prediction revealed that they were significantly different in their spatial structure (except for the conserved R2R3-MYB domain) and expression pattern, which indicates that they may respond to cold stress in different ways. These three key candidate genes may provide important information for genetic improvement and research on cold tolerance in *S. album*.

## 5 Conclusion

R2R3-MYB genes play important roles in the growth and development and abiotic stress of plants; however, there is no relevant report in *S. album* yet. In our study, the R2R3-MYB gene family of *S. album* was identified at the whole-genome pattern, and its characteristics were analyzed and its expression pattern under cold stress was studied. Three important candidate genes significantly related to cold resistance were found by combining transcriptome results and WGCNA results. These results fill in the gap of R2R3-MYB gene family of *S. album* and provide great significance for genetic improvement and molecular mechanism research to cold resistance in *S. album*

## Data availability statement

The original contributions presented in the study are included in the article/**Supplementary Material**. The RNA-seq data presented in the study are deposited in the NCBI repository, accession number PRJNA320980. Further inquiries can be directed to the corresponding authors.

## Author contributions

SX and LC designed this study and revised the manuscript. MT carried out the study and wrote the manuscript. LL and HX performed the analyses of MYB gene family and transcriptome dataset. HZ and ZW revised the manuscript. YL and QZ helped to improve the language of the manuscript. All authors contributed to the article and approved the submitted version.
