# Supplementary material for: Genome-wide characterization of R2R3-MYB gene family in Santalum album and their expression analysis under cold stress
Source: Front Plant Sci. 2023 Mar 2;14:1142562. doi: 10.3389/fpls.2023.1142562 (PMC10017448; doi:10.3389/fpls.2023.1142562)
Supplement: Supplementary file 1 [file DataSheet_1.zip › Supplementary-file-S2.docx]

**Supplementary S1. The protein sequences of 114 R2R3-MYB genes in S. album**

>SA01G00117

MKERQRWKAEEDAILGAYVKQYGPREWHLVSQRMNTPLDRDAKSCLERWKNYLKPGIKKG

SLTEEEQRLVIRLQAKHGNKWKKIAAEVPGRTAKRLGKWWEVFKEKQQREHKENHKAVDP

IEEGKYNQILETFAEKLVKERPSPAFLMVASNGTPPTLLPPWLSNSNGASTARPPPSPSV

TLSLSPSTVSASPAIPWMQPERLPDNALVLGNLPPGTGENLLISELVECCRDLEDGHHAW

AAHKKEAAWRLRRVELQLESEKACRMREKMEEIESKMKALREEQKVMLERIEAEYREQLA

GLRRDADAKDQKLAEQWTAKHMHLTKFLEQMGCRTRLSNPNGR

>SA01G00299

MGRNPCCSKEGLNRGAWTSCEDQILSDYIEAHGEGHWRSLPKNAGLKRCGKSCRLRWLNY

LRPGIRRGNISRDEEELILRLHRLLGNRWSLIAGRLPGRTDNEIKNYWNTKLSKKHQKPA

TDKGVDASKEPLATQLHTVVRTKASRCTKVVMDELHSSTGELDTNPSTNDIPIADNNILS

DFRMDFSDEKDQSSMDLLSYYGFSEFHNEFGYNHYGNYVDEYIGSSAPSIRSDGGNGIVL

PQEDMGLVVEDWIVNADFLFA

>SA01G00342

MGRAPCCDKANVKRGPWSPEEDATLKNYIHSHGTGGNWISLPHKAGLKRCGKSCRLRWLN

YLRPNIKHGGFTEEEDYIICSLYSNIGSRWSVIASRLPGRTDNDVKNYWNTKLKKKLLSG

TTAGVNSMNFIQSSQSSAELSYFHQMPTLPSPPVVSNLHTDGSNNVSGSSSVGLDSWGGN

GEDDELLMTLCFEASDHFLNGPVFEEQISEVHDPNLSNPPQC

>SA01G02184

MAGSIQRKEMDRIKGPWSPEEDGALQRLVEKHGARNWSLISKSIPGRSGKSCRLRWCNQL

SPQVEHRAFTPEEDDAIVRAHAKFGNKWATIARLLSGRTDNAIKNHWNSTLKRKCSSGSG

CDAGPSSPPLKRSVSAGAAIPFSGLNLDPGSPTRSDVSESSTSHALSAALDPPTSLSLSL

PGAGGACESTPVMEGRIPAEPEPGRPFVPFSPELLSVMQEMIRTEVRSYMSGVDRNGLCF

PAGDGVRNAAVKRIGVNKID

>SA01G02458

MARRTIVNASSSGNEVAGGAGGRKGMKKGPWTVAEDEVLREYVRKHGEGNWNAVQRNTVL

LRCGKSCRLRWANHLRPNLKKCSFSSEEEHLIQQLHYLYGNKWARMASQMPGRTDNEIKN

YWNTRMKRRQRAGLPLYPQGTQKEEPAPTFLPQRRHLQQQPSSPLISSSSLSDLEKPKLN

YNPPFSPLFSPMLNFSTQIPDTKHSLFPSINPRPELKLFDSINISNARAFSLPPMVLPSP

SLKLTMNPSMIISSSFESFSMIQRKMNVAELPSLQTPASPSAPAVSSESVGDYFLSQLLP

KGNSGLLDELLKEARALNKGEAKDNDTIESEVLQLDGSSSRLKKHRNVTLVEEMSSMDED

MLSLLNFPLSTFPDWCLETVDKPNEHSCGMKNMLSFASYDLRG

>SA01G02958

MGHHSCCNQQKVKRGLWSPEEDEKLIRYITTHGYGCWSEVPEKAGLQRCGKSCRLRWINY

LRPDIRRGRFSPEEEKLIISLHGVVGNRWAHIASHLPGRTDNEIKNYWNSWIKKKIRKPT

APQATSVVPNTDHSQLSYIPNQPNLSNQDSLTRCTTPTQETLIPSSCPLFMFDTGLMGGN

NGDNNSVQSDLFHTESATLNSETWSHLPQVNQLTLPMPPLTTSFMTSGPLYSSSLPPLIE

NMESLVDMPLEMPQCRSSVDIEEGEIITMNNSYWIDQASQCCPNFLFWDPLEIGGEEIAP

SDSTNMGTYLSTYTTPSL

>SA02G00494

MGRSPCCEKAHTNKGAWTKEEDDRLAAYIRAHGEGCWRSLPKAAGLLRCGKSCRLRWINY

LRPDLKRGNFTQEEDELIIKLHSLLGNKWSLIAARLPGRTDNEIKNYWNTHIRRKLLNRG

IDPATHRPINDPAAAGVDVTTISFVATAPSSEKDEQEKIARGFMKSPVRERCPDLNLELR

ISLPYQTQIDPLKTSRDWDCNSGSSSGGSIGYDFLGLKTAVLDYRGLEMN

>SA02G00631

MGAGYLDRINVPWSQEEDEVLRRLVLRHGVGNWSLIGGSIPGRSGKSCRLRWRSQLPPDV

EHRPFTAAEDELILQAHAQHGKKWAMIARFLSARNGDAVKNHWNSTLKWKFSPVIRMEDG

GSSGHGFRPEKRSASSDSEFSGSRQIDASSEADDPLTRLSLSLPGTESEEPSSREFVCSN

PIRLPRRASENSSPEFVALLREMIEEEVRNYQREHWERRRDQSK

>SA02G00740

MGRQTGSSKQKLRKGLWSPEEDEKLYNHITRFGVGCWSSVPKLAGLQRCGKSCRLRWINY

LRPDLKRGMFSQQEEDLIMSLHGVVGNRWAQIAAQLPGRTDNEIKNFWNSCLKKKLMKQG

IDPNTHKPLSESKPENDDFPENSSLQLPQTEPTTFLINNSGYSHAGLIEAATGEDFMEKP

EFDPSSFFEYEYSPDFLHIQGQQITSPVDQYHWEANSNSAGFNSMHSLSNFDQNRRLQPN

EFSDNSTSRMGSLFLNEGRESSSNSSTINCHSMVENVGFSWKEQGQNLHSLDADCFDGCF

LTSSSSEDLAGASLDVFQQV

>SA02G00757

MEGEYKKGLWTAEEDSILMDYIRVHGKGRWNRIPKLTGLKRRGKSCRLRWINYLCPSVKR

DEFSEQEDDLIIRLHNLLGNRWSLIAGRLPGRTDNQVKNYWNIHLSKKLGIRKRKLRIGS

PSESLSVQERDKGGQFSASPPGLRLDYSSPKSGLDGTTIGIEGENVKNIGSEHAAGFDGT

DDASIMNGSYGSSSWILNDDLGLCVPTLLEPLDDYSFDFIWNTF

>SA02G00807

MGRPPCCDKMGAKKGPWTPEEDLVLVSHIQEHGPGNWRAVPNDTGLLRCSKSCRLRWTNY

LRPGIKRGNFTEHEEKMIIHLQALLGNRWAAIASYLPERTDNDIKNYWNTHLKKKIRKLQ

TSDASHREGLISSSSSSSSMVSQQNPNNNSKGQWERRLQTDIHMAKQALLEALSLGQSTC

TSRDIQKPRAANHPYASSTENIARLLQGWTKERSSDAARVGSRVARNDSSNPASGIDSSF

INSSELTQKLSQSTSTGSEDDVALYDEREKHDKSSTTQLSLLEEWLLDDTGNVVGSLGQE

DLMDMVPEDTVQLSY

>SA02G01498

MGRSPCCDENSGLKKGPWTPEEDHKLATHIQKHGHGSWRALPKLAGLNRCGKSCRLRWTN

YLRPDIKRGKFSQEEEQTILNLHSVLGNKWSAIASHLPGRTDNEIKNFWNTHLKKKLIQM

GFDPMTHRPRTDIFSSLPHLIALANLKDFVDQHSSFSSSSSSSSSWESHAAKLQTEAVQL

ARLQYLQFLLQPPMDNQSALNSLPYSLQSSAVDDSLSLPFSHLPELQAPCSFQTPMPSSN

KENGEVLNDFGSVISNQGELVNSLNNSPWFPATSSTPSPPPLAVPQLPEAACSTTVTNFN

QGDNFCSGGGSSCGGGGGGTSFWPDLLFDDALFQEIA

>SA02G02355

MGTKNESEDRNMYEDQEDSPSHEEDSSGGNSGESNALKKGPWTAAEDAILVDYVSKHGEG

NWNAVQKHSGLSRCGKSCRLRWANHLRPDLKKGAFTLEEERRIIELHAKLGNKWARMAAE

LPGRTDNEIKNYWNTRIKRRQRAGLPIYPPDVCLRAFSENQQSQSISATGDEHLDLLQAS

SLEIPDLELKNLGHNHGYQSYPPTLLDIPVSRLTPGLGSSHGCSFMFPTLHPSKRLKESE

SILFPHLDHNNVGTSFSQYEDDAYDRIDHPFGLSSPCDPAPSTNHQSVLGAFSGSHAALN

GNSSSSEVKKLELPSLQYAESQLGSLDLRPPSPLPSLDSIDTFISSFPNGERKSNDCVSL

RSNGLLEAILYESLALKNAKSSNSCQQTPSNSVVVDSSSPEFCASKLGDASGETMSPLCH

SAASAFSERTPISGSSMEETRLGHEIKHEVADDAVLTRYHNERKQTERKIDLSRPDTLLG

LDCCWFGLGTSHGTMDRSVLTTDAVEVFGEDFGREYGSYEDNAPAPVSVNGPETTEDSIE

LQWDQSFC

>SA02G02428

MTGSCSEEEEQFFDTLEDISSASDLGSDDGPENTEFDSNVSDFLLGTCRYDEWNKNPASI

CERRNKFLKLMRLSSEKPARKDTEVSCYESKTKTNKTIENSGAVLGSSDFKDESLSSQSL

TSFWSTDAPQPLDGTSEKNLVCRVKSLDDGTDFTVDEPGLDGVLNGPSEVCSNQSVTNEE

FERNLSFSQLVQRVMRRSMVDVCNLPAGSKFGTRGWFRKLGIVDSQVETGGLIINKPCTM

AGVQTRMVKVRSSKKRFKEFPALYMGQDIVAHRGPILTMKFSPGGHYLASAGEDGIVRVW

QVLESERSDIFDNLDIDAASVYFTLNHHSEIVPLHFDKQKRSKLISLRKSSDSTCVIFPR

TVFQILEKPLYEFHGHDGEVLDLSWSKNKHILSSSADKTVRLWQVGCNQCLKVFYHNDFV

TSVQFNPMDDNHFLSGSIDGKVRTWAIPDCQVVDWTDIKEIVTAVSYFPDGKGAIVGSMS

GKCRFYNASGPRSAATQAPAVFTPDGSQIVLATEDSNIYMWNYSSEDDNVSGQGKNNWSF

ECFSSNNASMAIPWFGMMDGNSIFSSMPGKLHFPKLFMDMLRHTEENGSQQCHVTGSSCK

TVPISSPDHVYLSLSSKEMLPRSNSSVMPSPKSKTPYKFLCSCCHGIFCSPHAWCLVIVT

AGWDGRIRLRHLKIGWLPSSSSSSLKPCESSISKKEEEEEKGLIQTSSMGRAPCCSRVGL

NRGPWTVREDALLTNYIEAHGVGNWRSLPKRAGLLRCGKSCRLRWMNYLKPDIKRGNITP

DEDDLIIRLHSLLGNRWSLIAGRLPGRTDNEIKNYWNTHLSKRLLSEGTDPDTHNRLSDA

VREVKKRKSRSNRRRSNKKGEEDRPRTVRAESVDRERPTIYLPRPTRITSRVRIPWSSFE

DGTHESLGFATDGVGGLPWFDFKGAVDNEDDNGMDIFNGDDAKVGQFNSPDFVCQSPLSA

GDATLEKLYEEYLQLLNAEEDQAAQLDSFAESLLA

>SA02G02578

MGRAPCCDKQLGLKKGPWTADEDDILTSYISKHGPGNWRSLPKLAGLLRCGKSCRLRWTN

YLRPDIKRGSFTAEEEKLVIQLHGILGNKWAAIASQLSGRTDNEVKNLWNTHLRKRLQQM

GLDPQTHKPISTTCGIDSKSASARHMAQWESARLQAESRLSRESLLFSPTQAKADTTDDY

FLRIWNSEIGDSFRKLKKGSEMACQSPISQASSSTKCGSVSGITLEMGHNTGDPCSKDGN

EDENKHKSSYREGTLAESDSSSSNELEDSSDTALQLLLDFPINNDMSFLGHSDDYFSCH

>SA02G02658

MGRSPCCEKEHTNKGAWTREEDERLINYIKSHGEGCWRSLPRAAGLLRCGKSCRLRWINY

LRPDLKRGNFTDQENDLIVNLHSLLGNKWSLIAGRLPGRTDNEIKNYWNTHIKRKLLNRG

IDPQTHQPLLLNNPSSTIHARINSQIATNHHGSDNHNNKSSSNDSNKSQLDHQSPPTVTA

SSQQSATSVPHFNCATTTTTSGKSGTSTEEIFSEEVDLELSIALPRGPNTNGGDYQLDLN

SKGLESAVWTIETNESKNGPEERLAKQFTGDVSPENEQEPVCLCCPVGLKNARECSCKST

VMMTTTNMIAATDLHRYYRPLDL

>SA02G02743

MGRAPCCDKASVKKGPWSPEEDAKLKSYIEQNGTGGNWIALPQKIGLKRCGKSCRLRWLN

YLRPNIKHGGFSEEEDDLICNLFITIGSRWSVIAAQLPGRTDNDIKNYWNTKLKKKFIGK

QQQQLQQRRHREQQAILDGEIMMATARDRSLFAVTSTAEAMNPHLYWPPSDQLPAIPIST

TTTAATPSVDSAVIHEQFLGISNCVDFDLERFDDDDDDVVILNGSCNTATNTTTTGSSSA

ESNINFNWEDSNINTTSEHDFELPYYPSAVLSNNNNNVRYEAVHQQQQDCSFEEYFWMQ

>SA02G02808

MDRIKGPWSPEEDDALQKLVQTHGPRNWSLISKSIPGRSGKSCRLRWCNQLSPQVEHRAF

SPEEDETIIRAHAKFGNKWATIARLLSGRTDNAIKNHWNSTLKRKCSSIGDDGSIAGGSG

GYDAEPTSQPLKRSVSAGAALPVSGLYRSPGSPSGSDVSDSSVPVLSSSHVYRPVARTGG

IVPPSQSQHLETSSSTNDPPTSLSLSLPVVDSPEVSNRARGSSPMQLFPLPPVSSPPAPS

NHIPFHPDLGSPAAEKPFVPFNAEFFTVMQEMIRKEVRSYMSGLEQNGVCMHAADGFRSA

IGISKIE

>SA02G03385

MEGGVGSFWSQNKNLRNNGSSSPSSSTLIFQPSSRPLTAIDRFLCGQNHPKIHRQTKPGN

FRQGAHGCFDFSSLSPYGGGEAIHGGFSRPAHLVEATSFVDGLFLDGNNSLNWSYLMEEK

ENPKCGGLLGEKRKFVEMGSTCRGKEPKKGNTKGGSSSSSSTAYLIKGQWTEEEDRTLIK

LVKQYGERKWAQIAEKLTGRAGKQCRERWHNHLRPDIKKDGWSEEEERLLIAAHRKVGNR

WAEIAKTIPGRTENAIKNHWNATKRRQNSRRKSPKNPSSSSSSSSFSPPNTNNNSTNPKP

STLLQDYIRSKTLHFPSPTTTTAAVTIANSATTSTASGASEDNSLSTNDKQLNIFRPPEA

AAPEEDLSPPRVIPPEEDEELLFMQNIISDYSYCNSSCSNNNNNQWLNSEERYIADRTIP

QFPLMGSAEEAAPATHLYSDLYLSYLLNGGADPSLSLSSYSSSFVDYSGMNDTNSAELQQ

LRGMGEMDLIELVSSSSQFY

>SA03G00456

MVRAPCCEGHGLKKGAWTPQEDQILIDYIDKHGHGRWRSLPKLAGLNRCGKSCRLRWTNY

LRPDIKRGPFTPDEENTIIQLHGLLGSRWAAIARSLPGRTDNEIKNFWITQIRKRLPSAD

MGTNNMKSQSSATNHMVQWESARVEAEARLSMESSSLINHSSMRKTSSDIYLYLWHSEVG

ESFRKINRRAAVKSESPNSPTSSLAKCGASTSTGLMNQQSDSMECTSRAPTGVPAKDGSD

CLSSFKSVDSSDFTLELLLDSPSSDAGFSE

>SA03G00457

MGCKTLEKPRLKHKKGLWSPEEDQRLRNYIVRHGHGCWSSVPVNAGLQRNGKSCRLRWIN

YLRPGLKRGMFTLQEEETILTLHRLLGNKWSQIARRLPGRTDNEIKNYWHSNLKKKVTQV

EEQQPTNIHCADLDPNPNLIERLCSPRNSVSEVSSFESSLLSAEGFAVDVNVGQSIQQVF

GSPEDSCRTPLPKILFTEWLPSENVQNWSSFEDGFMDGSGRDGLENRSARDTVSVSLDHM

KLEDPSIIENGYGAFIPVGEELVWPFQFGHFN

>SA03G01026

MHKLGFAAQWIHLVMTRVATVADSLLINRKQSCCVATTIEAPVKSIHLLEDKVSSFGLNW

AKEFGCSSLMVESDSLIVLFKLCSNSESLPTLGHLVAERSVPVGSQMIPDGGTALNNDYL

QEPGGEGGGSIGGCDCGGDGGGGSGGGGSGGSLAPAPAPGRRLKKGPWTAAEDAILTEHV

RKYGEGNWNAVQRNSGLARCGKSCRLRWANHLRPNLKKGCFTSEEERLILQLHSKYGNKW

ARMATQLPGRTDNEIKNFWNTRMKRRLRQGLPLYPQDVQHSHGPSPRPLTPLSPSTPSLS

LSSPHQYLLSPHQSYHQSPTLSLSDHNATPFSFSSPLTPHSLPTLQGPLYRFGRLRNTVP

SNAHPITFSTSVTPSPSPMLASPLSTPLSQFSTPQLSPMSFEANSPPPTLQTQFQFDDMG

FPNLGFMAKSELPSNQLFQMQHFGGPEVERDGVSQHGNSSSGYLDDIFQEAQATVGIKAL

WRDDLDEKHVDDGFGSLISNNSSGHLVPTSVGGQWDDSSSVHYSAGAKSKEEPEEMIEFK

HEDLSDLLITFPLGMGSPEWCSHKNSSNEHSVGQSSAITDDSGELKMQQLSSSFPISDSS

ENGSCSWSYMPPMC

>SA03G01221

MGRIPCCEKDSVKRGQWTPEEDNKLSSYIAQHGTRNWRLIPKNAGLQRCGKSCRLRWTNY

LRPDLKHGQFSDSEEQTIVTLHSVVGNRWSLIAAQLPGRTDNDVKNHWNTKLKKKLLGMG

IDPVTHKPFSHLMAEIATTIAPPQVASLAPHHVANLAEAALGCFKDEMLHLLTKNRVDFH

LPPPKHEEKDDDNITTGTTTTIEKIKMGLSRAIQQEPVINKPWDSSSAGGPANFLACGAF

PGFQCSASSFGNDGTTSSWGQSMCTGSTCTAGDRNERLLAKLEDENEESEDAKTIKNRSN

IFGSDCDLWDLPSDDLMNPMV

>SA03G01226

MGKGRAPCCDKTKVKKGPWTQAEDIRLISFIQKHGHPNWRALPKQAGLLRCGKSCRLRWI

NYLRPDIKRGNFSPEEEQIILNLHCSLGNKWSKIASQLPGRTDNEIKNHWNTHLKKRAMP

SNSLSKSSSLASSPCDSSVSNGGIQRIFNKANSLESEQTSPSHEANPGLEHSNVNKGASS

VEEAIEIPFVDEDLWESIVYDDGEDYGNKEWSAKGSHEGTESWGWLEYLENELGLIGEAP

TTAGEIEINAAAVHEQPPAVMSTCLNQHQQEGLLSEPEFDPIVTYFHTWPTSPHDLLGF

>SA03G02211

MCTRGHWRPAEDEMLRELVERYGPHNWNAIAEKLRGRSGKSCRLRWFNQLDPRINRSPFT

EEEEERLLTSHRLHGNRWAIIARHFPGRTDNAVKNHWHVIMARRSRERSRFYAKIRAAHQ

MPLLMNGKKHSGKRFDAHETSGDGDEETKNNKDVASFVDKYCRYGRYGYPFSSHGHCGSL

KDLYYGKTSHGVKLHEDKSHQPIEFYDFLRVHTESNASEVIDQARSKEDEEVEQKTNMEN

HVPFINFLSVGTSS

>SA03G02236

MSLIDRITARAQSWTQRFLSFAGRLQLIKSVLFFIHVYWASVFILPKAIIRQIEQILRNF

SWKGLGLQESLWCQWTHSNFLKHKSFWDLKTPTVGYWEWRKILQLRPLVHWFLSWRLRSG

QDVLLWFDAWLPLGPPSHHFSNRAIYNLSIARNAKVAEVLSAGNFHWHVSLGLQSWSAAI

PSLDSMQNDTLLWNGNPAFSMAIVHDTFRGRDPDQNWARGINSPDSAKMRIMIKGGVWKN

TEDEILKAAVMKYGKNQWARISSLLVRKSAKQCKARGSSPAWYIKHHKTFPHPDVELSTL

AFMCWGNLGFARAQCKTQIGHPSGQKKGAVILCQGSTPKPTGNGTEWTREEDEKLLHLAK

LMPTQWRTIAPIVGRTPSQCLERYEKLLDAACAKDENYEPGDDPRKLRPGEIDPNPESKP

ARPDPVDMDEDEKEMLSEARARLANTRGKKAKRKAREKQLEEARRLASLQKRRELKAAGI

DNRHRKRKRRGIDYNAEIPFEKKPPPGFFDVADEDRTVEQPKFPTTIEELEGKRRVDVEA

QLRKQDIAKNKIAQRQDAPTAILQANKMNDPETVRKRSKLMLPPPQISDHELEEIAKMGY

ASDLLAGGEDLSEGSGATRALLANYDQTPRPGMTPLRTPQRTPAGKGDAIMMEAENLSRL

RESQTPLLGGENPELHPSDFSGVTPKKREILTPNPMLTPSATPGGMGQTPQIGMTPSRDG

YSVGMTPRGTPLRDELHINEDMDMHDSAKLELQRQADLKRNLRSGLTNLPQPKNEYQIVI

QPAPEEGEEPEEKIEEDMSDRIVRERAEEEARQMALLRKRSKVLQRELPRPPAASLELIR

NSLMRADEDKSSFAPPTLVEQADEMMRKELLNLLEHDNAKYPLDEKVDKEKKKAAKRSRT

GKSADFIPVIEDFEENELQEANHMIEAEGQFLCAAMGHENESLDEFIEAHGTCLNDLMYF

PTRSAYGLSSVAGSMEKLAALQNEFENVKTRMDDDTKKAQRLEQKIKLLTHGYQTRAGKL

WIQIEATFKQMDTAGTELECFQALQKQEQMAASHRIKGLWDEVTKQKELEKALQKRYGDL

MAEQERVQDLIDRYRADEQMQEEIAAKSRALLLAEAAANETVISGEAAPDHRAVDESEKS

SPVDPSHIEASDMDVKISDTKSSAAEDANENQPSAPVDDSVENRDGLPDPTVLEDNATVG

DSVEGMDGENLITTEVSKEDTSVENALDAKETQNMDGSTTEPGESVCETSLNGVGESLNG

VGESVNGVLNNSSEIDSSLPAS

>SA03G02255

MVIKGASCEKMRIKKRPWTPEEDKILISHIQQFGHENWRALPKQAGLLRCGKSCRLRWIN

YLRPGLKRGNFTNEEAETIIQLRHSLGNKWSTIASKLPGRTDNEIKNFWHTQLKKRAVRQ

EPMEPHSPPAIFMPEANSTIASSQVKALVSEEEPPTPNTYSPVTAGSNSPITDYIPAVAT

PSPYDAASSQDPAFWTGELSAFIFDFVNLGSFSCDIASGKLVKCTKRENPTPSDMIFLGI

TLSEYDSNEA

>SA03G02959

MKRQAMMMKKTGDEEPKQKERHIVTWTQQEDDILREQIRIHGTENWTIIASKFKDKTTRQ

CRRRWYTYLNSDFKKGGWSPEEDMILCEAQKVFGNRWTEIAKVVSGRTDNAVKNRFSTLC

KKRAKHEALAKENANSYNKTNKRVLIQNGDNTDITSDIAGSLKRMRRSHIPIYPNSGGNL

LGEYGNPVHQQMRSPFSVLVQNFHNVNSLAVHHHVDSVKEASNDAQSNKIQGTFLRKDDP

KIAALMQQAELLSSLAVKVNKENTDQSLENAWKALQDFLDESKESDMPRVKFSDMDFQLE

DFKDLVDDLRSNEQGPPSWRQPDLYEESSASSEYSSGSSLLSQTTADKMEQIQAEYCGVQ

QDTGVGLQTVDPGDQNGFDDCGNGIRSRTSTDQADIFLSCDESGNNEGVVSGISRTEFSS

PLQVTPMFRSLAAGIPTPKFSESERHFLLKTLGMESPSTKPSTNSLQPPPCKRALLGSL

>SA03G02998

MGSLSINPNHCGVASSSTLSSSQESYVSKNENEGLSSWGFPLIAQNCTIPNLEDSSDGGG

ENNEAFVPSVAGNSIEESPNEVMSSGKEEISCGQSKLCARGHWRPAEDVKLKELVALYGP

QNWNLIAEKLEGRSGKSCRLRWFNQLDPRINRRAFTEEEEERLMAAHRLYGNKWAMIARL

FPGRTDNAVKNHWHVIMARKYREQSSAYRRRKLSQSVYRRRMEDESTNGRGGLMSNLQSF

QFSSNDPPPQMAHMAFGEAILGTSFPPEQTSFDFFSGPKSHDMMRSLWSNNRPWAGPRNE

QISGFYAQTHTSLVVAMEQSNLYNPYYSFSDPTASNLQVSSSTEPSSSDTERSGLGGHYG

TNPPQFIDFLGVGAT

>SA03G03010

MGRSPCCEKVGLKKGPWSPEEDHKLLSYIEQQGHGSWRALPAKAGLQRCGKSCRLRWTNY

LRPDIKRGKFSLHEEQTIIQLHALLGNRWSAIATHLPKRTDNEIKNYWNTHLKKRLVKMG

IDPVTHKPKTHALSSSGAHYSKDAANLSHMAQWETARLEAESRLARGGSSSSFKPVMPSS

NLHYYSFPTHQLGSSLAQLLLNRTNTVAMPLPPQANTVHCLDILRACSIPPMLENTNSSS

VFSVPVGLGETMLGGNDSSVFYDDHTMRGMWIGAAGNEGISVPNLDSMTDSAMDHCGDWT

AVHRGVGGSCEEMGMSCGIEESIFNVEHFLEK

>SA03G03219

MSEIKEWSGWFADNKSHLPDLSPRASQKLQSCERRYFTELNGGQSLEFERQKIEGDGLRA

LKIQLEQIAACRHCSFTLSFKPSASTTLQISTLPSSKLTSTTASSSNHEATTPSTAFESI

PSGLPRSDLDARRDVLPVIKAIKNNLLGPFLDLVATKLNNTAPSVTCLVTDTFLSFPYAA

GEVLGFPVMTLCPAPACAIMGILQYRPLMDKGYIPLKGIHRSADPRPGDRYIGPFNLVAP

GTNSPAHGARRTNRSLDLMQPCPGLFVFWHKPAPLEPHSPCPSLGIFIISNWVESGFLLP

INCIENYSKNEMLIGIMPYGSLSLPTKSKSPNNSPSEEEGGSELRRGPWTPEEDALLIHY

IACRGEGRWNLLAQCSGLRRTGKSCRLRWLNYLKPDVKRGNLTLQEQLLILELHSKWGNR

WSKIAQQLPGRTDNEIKNYWRTRVQKQARNLNIDCNSAAFQNVLRCFWTPRLLEKIEGSS

SDVMSQNPTISPQPLTSHSTQHLSPQLIPQGPFPSVDDHTGTNSISANRDSRSLCSSESM

NVSQVPEIYGYPNCPIQAIRNNNSTDYSTIPRHDDMEGSLSMAYMSAVGDFGGPVGDSQM

VDGAWVENDLADSLWNIDESAILRASTRGSRRKILKDKNAEPTEFEESVAQNTYDELRSD

LKDLFINSAVQLDVSGGRKAVVIHVPYRLRKAYRKVHVKLVRELEKKFSGKDVIFIATRR

ILRPPKKGSAVQRPCNRALTALHDAMLEDIVLPAEIVGKRTRYRVDGSKIIKIYLDPRER

NNTNYKLDTYAAVYRKLCGKDVVFEYPIMEAWNH

>SA03G03233

MTLGRLLAQPSYAPAKPYTPLKALGEEREASKMRIMIKGGVWKNTEDEILKAAVMKYGKN

QWARISSLLVRKSAKQCKARWYEWLDPSIKKASTLLYECKFSLRDCWLLIVSLYLEYTST

EWTREEDEKLLHLAKLMPTQWRTIAPIVGRTPSQCLERYEKLLDAACAKDENYEPGDDPR

KLRPGEIDPNPESKPARPDPVDMDEDEKEMLSEARARLANTRGKKAKRKAREKQLEEARR

LASLQKRRELKAAGIDNRHRKRKRKGIDYNAEIPFEKKPPPGFFDVADEDRPMEQPKFPT

TIEELEGKRRVDIEAQLRKQDMAKNKIAQRQDAPSAILQANKMNDPETVRKRSKLMLPPP

QISDHELEEIAKMGYASDLLVGGEELTEGSGATRALLANYDQTPRPGMTPLRTPQRTPAG

KGDAIMMEAENLARLRESQTPLFGGENPELHPSDFSGVTPKKKELLTPNPMLTPSATPGG

MGQTPRSMSQTPRIGMTPSRDGYSFGTTPKGTPIRDELHINEDMDMHDSAKLELRRQADM

RRNLRSGLTNLPQPKNEYQIVIQPSTEESEEPEEKIEEDMSDRIAREKAEEEARQQALLR

KRSKVLQRELPRPPAASLELIRNSLMRADEDKSSFAPPTLIEQADELIRKELLNLLEHDN

AKYPLEDKMDKEKKKGAKRSGSSKSASFIPVLEDFDENELQEANHLVREESQFLIAAMGH

ENESLDEFVEAHRTCLNDLMYFPTRSAYGLSSVAGNMEKLTALQDEFENVKKRMDDDTKK

AQRLEQKIKLLTHGYQVRAGKLWTQIEATFKQMGTAGTELECFQALKKQEQLAASHRIKG

LWDEVTKQKELEKNLQKRYGDLMVEQERIQEIIDNYRAHAQMQEEIAAKNRALEYAEAAA

NEAVGSSTVDESGRSDPVDPSHNETPSQQIESTDDEQAYASPEDGMHNDTKEVLTALDTD

MMISDTKSSAAEEAQAANENYTNSTPAHGSLDNGDMLLGPAVVGEVAMMDGSVEGTAGEN

LIGSEVPKEDTAVENVPDVSEAGDMQGSMTEPEDVIKEPLGDGKNGNGESMNGVVAHASG

VEPALPVLDTSDTDTGKEP

>SA03G03257

MRKPCCENRETNKGAWSKQEDQKLIDYVQKHGEGCWHTLPKAAGLLRCGKSCRLRWVNYL

KPDLKRGNFQEDEIELIIKLHGLLGNRWSLIAGRLPGRTDNEVKNYWNTHLRKKLSLMGT

VPKHHHHHHHHHLHLSGGVAQCHNNTNNNIDSNNRSNNSNINQCENTASPERSPTPISTG

TGTGIPDLNLDLTLSTPPPAAEEHRTHHACNRDIDTGTPATMLLLG

>SA03G03312

MTPCCERKGMKKGPWTPKEDEILISHIQQFGHDNWRALPKQAGLLRCGKSCRLRWTNYLR

PGIKRGNFTEEEEETIMKLHQILGNRWSAIAARMPGRTDNDIKNLWHSQLKKRAVKQEPQ

GGHPPPEMSMHQFSNAVLPSHHHHHHQWNNLSTMGNNSVIKEEPSVCRSIMSNNSPTIAT

AGSYNFPITADLTMASVIQVPPAYEYGWEAVQCNKGLGAVTEDMIFSIPWSRRVVLVSAS

AAGSSNPSSDSNPYAVLGVNPIMGFDAVKAAYRRKRKEAESRGDEATAALVSKDIKYADN

QPILPWGPRFTKSSEKDMRINMAISAVFIAWILIKSNAEWKPLQFLGFVFVYRIFEKLKA

FEPPVSPTFTEEGGEDEGRMLRMGKRLLRSLALVFGCIAVSSLAYTGVLNLIEFVGSYIP

AFLYNNQCKMVTKSVDLRSDTVTKPTDAMRAAMANAEVDDDVLCTDPTAHRLETEMARIT

GKEAALFVPSGTMGNLISVLVHCEIRGSEVILGDNSHIHIYENGGISTIGGIHPKTVENN

KDGTMDLDLIESAIRNPNMELLFPTTRLICLENSHANCGGRCLSAEYTDRVGELAKKHGL

KLHIDGARIFNAAIALGVPVHRLVQAADSVSVCLSKGLGAPVGSVIVGSKAFITRARTLR

KTLGGGMRQIGVLCAAALVALNDNVVKLEQDHKNAKALANGLNKIEGLKVDISSVETNIV

YVDIEEGCKFTAHKLCKNVEEHGVLSIPECSSRIRFVLHHQISESEVQFTLSCIREAVSG

APVENGE

>SA03G03555

MGRHSCCYKQKLRKGLWSPEEDEKLLRHITKYGHGCWSSVPRLAGLQRCGKSCRLRWINY

LRPDLKRGAFSQQEEDLIIEFHAVLGNRWSQIAAQLPGRTDNEIKNLWNSCIKKKLRQKG

IDPNTHRPLSEIENEEDKLCSNNSNNERARSSASNEANLMIIEEENDRNSMPPPLAVSSD

DPNILSSSSSRGVIVVPPPTHQDSILDRPSELVGHFSFQHLNYGNDFGLSLNPVSSVLCY

NHMSRSSEMISEFCSNIMPNPIIPNSTQSLACSSVSGWEGSTLNHSSSSSNENNHSNELP

GNSSSFEENNGLMWVKSGKQTQFHHLLQPDPETNTKWPEYLHTPFLLGSALQNQFPQPSF

HTSERNKAETEFTVTDGSSTGTWHPNQEQQQDLQSSDIYHNKDIQRLAAAFGHIF

>SA04G00233

MGRQPCCDKLGVKKGPWTAEEDKKLINFILTNGQCCWRAVPKLAGLRRCGKSCRLRWTNY

LRPDLKRGLLTESEEQLVIDLHARLGNRWSKIAARLPGRTDNEIKNHWNTHIKKKLLKMG

IDPVTHEPLNKPNEAPPHTTHPLPESGDGSPETMPPATDNNVDMVNSDTNSTSTAENCSS

DESFLTDPIMSHLWVDDAPIIDGSWRFPSATEDVSLWENCAWLWDCEDFGVHDFGFNCLF

NDNIDTLEIGEHKH

>SA04G00800

MGSLSLMEEAAPMNTCTSGASSTSSESESSYTECTQAAERIKGPWSVEEDRVLTRLVQRY

GPRNWSLISHHIKGRSGKSCRLRWCNQLSPAVQHRAFSSAEDQVILAAHARFGNRWATIA

RLLPGRTDNAVKNHWNSTLKRHLLIHATAARENSSASGSGTGTSNAGSDLDPITALSLAP

PGTGTATAEEGRGQQRPETVPGEFWDAMRSVIAAEVKNYVTATFSEAAASSSSSSSGFRL

>SA04G00965

MPNRGGQKAIWAQKTGYMGRHSCCYKQKLRKGLWSPEEDDKLSRHITEFGHGCWSSVPKL

AGLQRCGKSCRLRWINYLRPDLKRGSFSQQEEDRVIELHAVLGNRWSQIAAQLPGRTDNE

IKNLWNSCIKKKLRHRGIDPNTHKPLWEVAGNKNNGTSLTEEENSNPPLVAADISSDKIN

DTRKNNSGTIFPPPTQEFFLDKSSESAGHFYFQQLNKGNHFSFSSVPAVCCSEMSRPSQM

IYEFSSNIMANANIPNSSLGSLTISGVQNWEANTFNRRGGSDSDTNGNNELHGDSSTFEK

SVLQWGKPGKQTEINEMEGDLETNNKWYDYLHSPFLVGSAIQNQIPQSSVYIDTTRDGTW

HQNQQQQPLQASDICNNKDIQRVAAAFGHIF

>SA04G02480

MMMMNYESIGAAGSGDDSRSCPRGHWRPAEDEKLRKLVEQYGAQNWNSIAEKLQGRSGKS

CRLRWFNQLDPRINRRPFTEEEEDRLLTAHRIHGNKWALISRLFPGRTDNAVKNHWHVIM

ARKHRERFKLSCKTSHKDAPNNSMKAQGQHKSASKLLESQATNKYGIFSLSPSCSASSWP

SCLPIEKNNCIVESSTSSDQSSYRLFSNPYPRHDYYYSRFIDGDGARESVKGDRLLLRSE

DDYSKNSLAMSATVQQERSRDKSVEQEDTPFIDFLGVGIPS

>SA04G02702

MGRIPCCEKEHTNKGAWTKEEDLRLISYIRLNGEGCWRSLPKAAGLLRCGKSCRLRWINY

LRPDLKRGNFTLEEDELIVKLHSLLGNKWSLIAGRLPGRTDNEIKNYWNTHIKRKLLRHG

IDPHTHRPITGSSAAAASPPPIPATEIALAMPSPEEGNCRSSITEEEEAFTTRNQRAIVD

LELSIGLSPFQSKPKPKPKPPQTTPHSWFPSESASGQTPFAAVTDTDTGAGAVRGGVCVC

CNLATGGGSCRNCGGPRGEYYRYCHSSFSLDP

>SA04G02784

MGHRCCGKQKVKRGLWSPEEDEKLTNYITAHGHGCWSSIPKLAGLQRCGKSCRLRWINYL

RPDLKRGSFSAQEEEIIIDVHRILGNRWAQIAKHLPGRTDNEVKNFWNSCIKKKLISQGL

DPKTHNLLSSHKRSSKNNYKPHQQQPMSPFSLIESPVSENQSLSQVWTMGGNNNGTMINF

PCTSSSINFNNNGTVPIPSSSSCSSLMNNPCGFGDLEEDCCIWATDNIESFGEALEEEVQ

PPAVEEEKLQNCELVGMDNDVYKEIDASFGSSTTTTATTTFNLDHFVESTLMSPMDIDLA

WNFQSL

>SA04G02814

MGRAPCCDKNQVKRGPWGPEEDLRLMSFIQKHGHENWRALPKQAGLLRCGKSCRLRWINY

LRPDVKRGNFTREEEDSIIALHQSLGNKWSKIASHLPGRTDNEIKNVWNTHLKKRLQLTS

KYSNHSDINNSPLGGKECSVSSSSSSTHTTFFCSKIRHEPEFRNLDPGHTLSKKEELMED

PRAIESDRPVIQTVEEVRKEPSSPSSSSSISPNLSKTMGLDLDLGLGQVMEPGQEGIPLE

SDLDFWDMLDSLGCGSCSNSTEAQSREVRASEECIINMRISEEDEDYRRWLRHIESELGL

LSDAEQPMANCGSPMPWPCPPPNFAI

>SA05G00189

MGRAPCCEKEGLNRGPWTPEEDRLLIAHIQNHGHGNWRALPKLAGLLRCGKSCRLRWMNY

LRPDIKRGNFSEEEEDSIIKLHEMLGNRWSAIAARLPGRTDNEIKNVWHTHIKKRLPWNQ

SSNLNPNSNLNPNISPAAESSANNPPFVAEPARGSGNSVTSLHPATPPQFESSSAGEFSS

VNDTGSSYTRERGPRGDEADEPSEDSVSFPPKMGQGFWSGAAESDFPAGELQFLQCFPFS

PLRVGEVPDCEYSACGESGDIGFLTNLFRDGELPLDSPEF

>SA05G00215

MDESWRKGPWTAEEDRLLIEYVRVHGEGRWNSVARVAGLRRNGKSCRLRWVNYLRPDLKR

GQITPLEESIILELHARWGNRWSTIARSLPGRTDNEIKNYWRTHFKNKAKDSSTSNSEEK

ARTRLLKKQQFQKQQQQFNQTGTKKITPLAAESENRVWTLTQMRNETSTMCPSPIEDQGF

SSTYPILSSEMCVPEAMNEENVLWDGLWNLEDIHVALMQQPVQ

>SA05G00504

MGRAPCCSKAGLNRGSWTAREDALLTNYIRAHGEGNWRSLPQKAGLLRCGKSCRLRWMNY

LRPGIKRGNIMPDEDDLIIRLHVLLGNRWSLIAGRLPGRTDNEIKNHWNTHLSKQLLSQG

TDPNTHKKLLDPVQEPKRRCRRGNRERSQKNSESTSEARTAKSNQRPKVYLPRPTRVTSF

VPISRSDSFESNGEGEPQSPGGEHGSGTEGVGVFPWSSLANGDDDAKGIDFLTGDCAADL

INGSDLGCETHPNMVGNPLQKLYDEYRQLLDTGESS

>SA05G00848

MPTKALCNSEEESDLRRGPWTIEEDNLLINYISQHGEGRWNLSARCAGLKRTGKSCRLRW

LNYLKPDVKRGNLTPQEQLLILELHSKLGNRWSKIAQHLPGRTDNEIKNYWRTRVQKQAR

QLRIDSDSESFLHAIRCFWTPNTLQNVEQVGPSHRPTFPFSSTLDSTISPPSISNHTPPF

NTAQGINHAEDNSGVLSSHQLESSQFHQISEHPASPCAYGNIFYNHPSLSGNLPMLDNSS

YDMEDYDPAAMSAIGNGGFFPSDCHMEEGDWASGEMEDTTWNMGELWQFGGVE

>SA05G00898

MGRSPCCGEIGLKKGPWTPEEDRKLVDCVAAHGGPGNWKSLPKLAGLNRCGKSCRLRWIN

YLRPDIKRGNFSEEEEAVIINLHSSLGNKWSKIATYLPGRTDNEIKNFWNTHLRKKLLKM

GIDPVTHRPRADLSFLGNCDISVLIRLLQAEATQLAQAQFLQNILRANSTISNQDSTGLD

QYKGLIGSYGSNNKLNSYLRVQETSTMNCADPLRSLLGLSSPIFIVPKPAAEEQVSNNNL

SDEFRGWYDILEENPSLPALFSVSDLETSQLVINEMGSTNVDKTSFSEVFESCNNWEKLM

DDGNSGSFWEGL

>SA05G01206

MGRTPCCDKNGLKKGPWTSEEDLLLTQYVQVHGPGNWRALPKDAGLKRCGKSCRLRWTNY

LRPDIKRGRFAFEEEEAIIQLHSVLGNKWSAIAARLPGRTDNEIKNYWNTHIRKRLLRMG

IDPVTHGPRLDLLELSSILGRNNPSDLNTLSSLLGVQSSPPPINPEWYLTLANTILSLKQ

RDPEILLRKIQQENQILLNSQLENQILSSLQFQPNSSFPIATNAPASATTSTAPQPNVEN

SSQLQQHSQENLIPSNWNADFQSLEIGNPNFGFDSVMSTPLSSPGQVNSSTAIRHSGTED

ERESYCSDLLMKFEIPESLDICEFL

>SA05G01212

MVRMPCYDENGMKKGAWSEEEDNKLRDYIQRYGHWNWRQLPKFAGLKRCGRSCRLRWLNY

LRPDLKRGNFTNEEEDLIIELHEKLGSKWSVIATKLSGRTDNEIKNYWHTHLKKRVGRNT

ASPTCVKEESSSTSECEAKHTEEEMLESVDTHLDTAVSTQQSSYGALSFELSDYGIWASM

DLAMEDSAGSPEMLTESFGSDFWTQPFIEDNFYMHNSGFGSQALLGGREFTSPYSAHYDD

DVDVFGRWMP

>SA05G01292

MDANVMSVGGCYYSSNPASYEAEEEMGDIRKGPWTAEEDLLLTNYIHIYGEGRWNSLARS

AGLNRTGKSCRLRWLNYLRPDVRRGNISPQEQLLILQLHTRWGNRWSKIAQYLPGRTDNE

IKNYWRTRVQKQAKQLKCDVNSKQFRDTMRYVWIPRLAERIRAESGSSTGTATQPMGSTN

NDFIGHPNYHYKSDGSSESSSEVTQVRRPDLCMTDGYNIGARVQYEPKSACGPEEFGGSS

PSGGYGKEEMDLDYPCLNLEQSDISDIGWLAGEDSSTKDTDNMTWNNEEIYDDLDAWLGD

E

>SA05G01520

MGLKKGPWSPEEDHILINYIRLHGHGNWRALPKLAGLLRCGKSCRLRWTNYLRPDIKRGN

FTREEEETIIQLHEQLGNRWSAIAAKLPGRTDNEIKNVWHTHLKKRVPKQQHADAAAAAA

ETFIFDNYFIEEEPKKNSSADNEESSSSESPRNHHPFLSPQPSSSDERSSITNAGRNNSR

DTNNLHVNQVDSPDGFPKMDEEFWSEVLSADTSTAAAEAPAAALAGDGVELEFSLSDQSL

SMELPAVCDEMAFWYDLFAKAGELPEFSEF

>SA05G01616

MHKMGKTPCCDKNGLKKGPWTAEEDQKLIDYIHEHGHGRWRTLPKYAEQGESGVGFFVWR

KKCIYGAGLQRCGKSCRLRWTNYLRPDIKRGKFSMEEEETIIQLHTILGNKWSAIAARLP

GRTDNEIKNYWNTHIRKRLLRMGIDPLTHGPLLELSSPAVGPIYFEGPSSYEDHNNVPSS

SNGIVLNEVLQQQPFSNLSNLSSSNSELIPQFFPQTLHHQQQNQMITFPNNHLQLPLSFQ

NLSSQDFQGRNATFSSSNASSSSSSSAHPFSICNGRSEVQQFSNPSNAVLNMDNDVSYSS

CLTAQNVSFPPYHSPTPSYNHMVFESSAAANNMMVGSDFGFGSGGSSLSSNMSYFNSSSS

DRAPSSFNGRTTPADAATDPYCNNMMVFDHYL

>SA05G01680

MMIGGSKRDLDRVKGPWSPEEDDLLRSLVEKHGARNWSLISRSVPGRSGKSCRLRWCNQL

SPQVEHRPFTEEEDDIIIRAHARFGNKWATIARLLNGRTDNAIKNHWNSTLKRKWMSISA

AEDDRDVFGGGGHQPPLKRSASAGPGYANPGSPSGSDLSDSSLPGAGSSSQFVYRPELRT

DPIALPVQQMQSTSGPTNEGPSTSLTLLPPGSDPSEVSSDPLPGSGHVEAHPPLQASESA

AQRRKSADSEKPFFSDEFLAVMQEMIRKEVTNYMAGIENDGLCSQTAEAFVRNALTRRIG

INKID

>SA05G02101

MAMVKSEEDFKVDVSKEVGSSISSSFSDSSYDTSTLRSASVQRRISGPTRRSTKGGWTDE

EQRKLVLDVHSLVQGHMIGLTEYFHGRSDVQCLHRWQKVLNPDLVKGPWTKKEDDCIIEL

VEKYGCKRWSVIAKYLPGRIGKQCRERWHNHLDPAIKKDAWTKKEEEILCFYHEIYGNKW

AEIARFLPGRTDNGIKNHWNCSVKKKLDSYSTCSSAQGVHGTMSPELYSHDTRADRSKVE

NLREISSRITPFTQDRASVRGVDSCSTDLVLGNANRGNGRLGTKPLQKSAVDEKLGSGHN

LNICSIDLVLGTGSGGTGCLRSKSVSLENGISSKGGINRIVNPLQLQFEENAVTATMLAS

KPCKNNAFDHSKMFDPVFTSLKDVSNSARIDHAEVVTPSTFGRIFESPKSFDSDAIGLTL

GSKLDSSFSSVSTSGFGKYSKSDSSFSSVSNYGFGKSNLGVSKRNQVYEPTPLNDSARAL

GNDSFPGTDNQKHPESPFPCSTPPSLVYNIPFKEGSPESILRTAAVSFNNTPSIIRKRTY

RKLNSKDVHHTGIVDARQGFHCVHAPEASIVVDSLKRNLEHSFDMERDFARGKLASQLLP

LGIP

>SA05G02124

MIDYGSTGAAGSSSGAGDDARTCPRGHWRPAEDEKLRRLVEQYGPQNWNSIAEKLQGRSG

KSCRLRWFNQLDPRINRRPFTEEEEHRLLSAHRIHGNKWALISRLFPGRTDNAVKNHWHV

IMARNNRQRSKRPCKRSYKDFFASSVKPNRNDKLLEFQETIPCSANSTGSNNNCIMENST

SLDQHPLYRYFSNSYRRHETLEFINGGADNCHSKNSFAVSMMRGMVRKEGGDDGCVFERK

DMPFIDFLGVGISS

>SA06G00100

MKERQRWKSEEDAILRAYVKQYGPREWHLVSLRMNTPLDRDAKSCLERWKNYLKPGIKKG

SLTEEEQRLVIRLQAKHGNKWKKIAAEVPGRTAKRLGKWWEVFKEKQQREHKENRKTGVD

PIEDGKYDQILETFAEKLVKERPSPTSFLMATSNGTFHHTDPSGPPPNLLPPWLSNPVGA

STVRPPSPSVTLSLSPSTVAASPAIPWLQPERGLDNNTPALGRLHEGLLVSELAECCRDL

EEGHRAWAAHKKEAAWRLRRVELQLESEKGCRRREKMEEIESKMKALREEQKVSLERIEA

EYREQLAGLRRDAEAKEHKLAEQWAAKHGRLSKFLEQMGCRPRLAEPSGR

>SA06G00181

MGSRREEERNEKIIRGLMKLPPNRRCINCNSLGPQYACTNFWTFVCITCSGIHREFTHRV

KSVSMSKFTSQEVESLQKGGNQRAREIYLKDWDAQRLRLPDSSPPYDYQYEDRRYGKQAA

VLTRKPGSDRGLYEGKISSFLYSPGRFSDQMCEDRFANESSVSRVSDYSVSSGGDTFRSE

AQSPNLQDTGISSPSNHRPGYISTELIQCQTLNTSSETKSGREADGIPPPQRTASLGSIG

SVDSNSVPPKSVNYDNSVDVVADVKQFSGVNQDTKSSFPTLPPSSVPWNPAGLDLLEAPF

APGPVSSASSANLFQPSETLSVQSVDFFQSPQISSAASMHLYSPPQNSSEISVPKDEGWA

TFDLPQSSASCLGNEIPTTGRTPSSDGNSLGNAHLGLSSNASMLQPSFQSSNAQGLSSTA

FNPWHSDLHKVQDFTGASINSWNAFEDSTSYRSVGNNQQISYSQPASGPSLAANPLGFKV

AEDPIKDVSQRDVVHGVPPDSRGGSFVARSSYTQPDNPSMDGTQIHASEHKSTNPFDLPY

DSEESSDLFLDMSSIQSALPASQLPPSFLGGVSEPWFPQNPATTYIPAAPQGGLGYIGGQ

TPASQLPLTQIWGERVKKKKWAMGRPPCCDKVGIKKGPWTPEEDLILVSYIQEHGPGNWR

SVPSNTGLLRCSKSCRLRWTNYLRPGIKRGNFTAHEEGMIIHLQALLGNKWAAIASYLPQ

RTDNDIKNYWNTHLKKKLKKFQSALEPHVASSHSSFNSSFSSKILTGTDTTDIASAATSL

ADQNSSIYASSTENISRLLEGWMRASPHPNQDKKRFLHEDKNLANYEGASRGISHEELDS

ILSFENMGGSLWDKSEKREGDLVGSERKMKLENNSPPLTVLEKWLLDEMGSTQVEEIMEL

SSPPSPPPPPMF

>SA06G00185

MGLEISTPEQQNEALELQLWEGEKKELTLKQQGSLGAARFEGLSTKLCSRGHWRPAEDAK

LKDLVSHFGPQNWNLIAERLEGRSGKSCRLRWFNQLDPKINRMAFSEEEEERLLAAHRLY

GNKWALIARLFPGRTDNAVKNHWHVVMARKHREHSSNVYRRRKGFNSQRGPPQTNKTPPP

NDSVNINYSSNNGGSESTISSNVDESVVSTSTELSLSSITAAAPPLGLTTGPGPILCHQH

YGPPRSGSRRERSMTMSDGYGLYGSEAMGKDESGHSNLKLAPNNNNSKNINFIDFLGVGA

T

>SA06G00186

MSSNLLGQQNEPWGIKSLQNMGVKTHDQEGNYGNPSTKKGLTTLSKGKKEEGGGLSVKRN

PTIRRCSKGHWRPTEDAKLKELVSQCGPKNWNMMAEHLQGRSGKSCRLRWFNQLDPKINK

AAFSEEEEERLLTAHRMYGKKWALIAKLFPGRTDNSVKNHWHVTISRRLREQASASLKNN

ASSLDSVLTRFEGSESATNDAPTESTFMCTDLSLSTSSKFSPSREHHGSQTSSFLSDLPC

QRQMRRAMGVNECNHEDLNKRVPAIDSVSNNRNLQMTGENEKIKMPIVFFDFLGVGAS

>SA06G00253

MEEDTVAVVDGEGGEGGGAATEACGVDGGGSGLDLVAGEWGGERRDEGEEGKGSRDRVKG

PWSPDEDAILSRLVSKFGARNWSLIARGIAGRSGKSCRLRWCNQLDPVVKRKPFSDDEDR

IIIAAHAIHGNKWASIARILQGRTDNAIKNHWNSTLRRRCIELDPTKLDSYNVVEEVSLD

KTKASSEETLSCGDASSARSLDGKDVTSLETFINQYEDRTQADYHFSHIGNDPTTLFRPV

PRVNASTVPMPNPDGGIRKPYVERSVPHQCGYGCCSTQGTRNAQKKSLLGPEFVEYSDPP

PISGHELAAIATDISNVAWVRCGLENSSVKAMEDAAGRILSHHHGSVVQQKGDVEDGTKK

NEHVRFEEGKNKLFGMMSTRVAGQTLPLPAKVGGSS

>SA06G00298

MGRSPCCSKVGLNRGAWTALEDQILTDYIQSHGEGGWRNLPKRAGLKRCGKSCRLRWLNY

LRPDIKRGNISVDEEELIIRLHKLLGNRWSLIAGRLPGRTDNEIKNYWNTNLGKKFKKPP

SAAVATAAPQQLPVRTKATRCTKAVGEMTNVTRSEPVTNNVGAATPVRDNAIPVWEDSIH

PLNFEAGLKSMEDDLFLTDFLNYSDFSDIYREFDCQNSDDNAQDLLGSSPPSICSDDSPG

IMLSQRDMETTLEDWIVDADAFLFSLGGNSNY

>SA06G00724

MGRAPCCDKANVKKGPWSPEEDEKLKAYIEQNGTGGNWIALPQKIGLKRCGKSCRLRWLN

YLRPNIKHGGFSEEEDNIICNLYISIGSRWSIIAAQLPGRTDNDIKNYWNTRLKKKLLGR

RKQSRRNGSTRDLKDANGEEEEEEEGGTSGSNYSPALSSSALERLQLHMHLQNHPFSLYS

DTAQWPKLLPLQEKMIQGLQFSVINEDPNPLHAQPLGMLQQQANDKLENPLNGMMSSDGS

IAFACEASNPLNSGSLPSSNCSLEQSNHNAGLGQQCSTFQPEFVLEDFLDHKAIEFRPQD

ANFRDELGYSRGVMGSSSSSFSDNVWWSSDELGSKSSTSSSWGTISCNGLDQCEGMFQDY

VLGYDML

>SA06G01020

MDPHYGMEISPTDSPLKKGAWSPEEDQKLIAYIRRYGIWNWTLMPTAAGLCRSGKSCRLR

WMNYLRPGTKRGNFSKEEDETIIELHQKLGTRWSAIAASLPGRTDNEIKNYWHTHLKKRL

EDSNNNSSQITSDFSLHISAPISPASPGGSCASTRNINGCSLDFTQLQSVGCNPFTMENF

GYWLEDQNHETTFGDSQFGLPTLQELQEPTYSHGSNNFGCCVEDQNQETTFGDSEFGLPT

PQGQQGPMYSYGSSTFDEEDELWLKCLIDADMTGISWPLYS

>SA06G01558

MGRAPCCEKVGLKRGRWTAEEDEMLTNYILANGEGSWRSLPKNAGLLRCGKSCRLRWINY

LRKDLKRGNISVEEEDIIIKLHASLGNRWALIASQLPGRTDNEIKNYWNSHLSRKIYSFR

RTSAPSSDDPLPAFIAEMAKLRSASKRKSQNIKSRVKKTEDSKKSPETMPLVPPTPTISE

GKILGKTGEDGEGEILGKTGEDGEGERGHFGSGGEGIDGELLCFYDIMSGDNTLMEASGS

LTPSVKGVENSRPILSESTPALENTINSLSLSNDGDRNDDGLIPGLSSTANLDFKDGMMM

DWDWDDNNNNSDDHGLWEDMFSWLLGQ

>SA06G01683

MVRAPCCEKVGLKKGPWSPEEDRVLVNFINLYGHDNWRALPKQAGLLRCGKSCRLRWTNY

LRPDIKRGNFSREEEGTIIALHQQLGNRWSAIAAKLPGRTDNEIKNVWHTHLKKRVLKQP

AAAELSANTSNSSSNSMQEENPGKMSSSSNEEASESPQQSSSDETSSSTNTKVDLPEDFP

EMDEEFWSEVLSADTSGTAAEASAVAGDTTGLEFAFFDELLTMEPVGSDGMEFWYDMFTR

AGEFPELWETDSSDWGQFIDSSKGQEINNFNRET

>SA06G01774

MEGMTREDVDDLRAAVDGDEAGGDGDEAGGDGGDGVARGRVKGPWCPEEDAILSRLVSKF

GARNWSLIARGIPGRSGKSCRLRWCNQLDPSVKRKPFSDEEDRIIIAAHAIHGNKWASIA

RLLPGRTDNAIKNHWNSTLRRRGMELGSIKPESCDMLIDGSHERTRTSSEETLSPGDSQS

FKCLMGQDLSLTKTQPGQYENKSQRKDHCVVETKDTSTLPRPVARISAFSVYNPPTTGSS

SSGAVPVQGPLVQHSKPGFGICNFFEGACAEPMVPSQCGHGCCSAPTKTHSQGSLLGPEF

VDYVDPPPLSSHELISIATDLNNIAWIKSGLESGSVRVPENASSWQPPEGAQTQTDISEL

MMKEGQGKLVGIMPEAASTQMPMQTFAFPAEVEGLS

>SA06G01827

MESSGGDRIKGSWTPQEDASLMKLVNQHGARNWSLISSGVPGRSGKSCRLRWCNQLSPEV

QHRPFTPAEDAVIVQAHALHGNRWATISRLLPGRTDNAIKNHWNSTLRRRRRAAATGIES

NSNGSEESGSKRRRLDPLSTEEGVVAGPATLLTLLPPGQIRRVVEEEDGGVSVKTETSDE

EEGDAMGEGIEGLDSDGKIENNGRKEEGSCLLKIMQRMIAEEVKNYINVLRAQDGVDFGL

GSKLESGARREK

>SA06G02089

MSQTTNESEDGMMSKDQTDSPLADEGNPESAGGGIALKKGPWTAAEDEILMDYVKKHGEG

NWNAVQKHSGLFRCGKSCRLRWANHLRPNLKKGAFTPDEERLIIELHAKMGNKWARMAAH

LPGRTDNEIKNYWNTRIKRRQRAGLPLYPPEVCLQAIQEGQQNQNADGIHVGDSGHSDLF

ETNSYELPDVTFNGLKANQVVFPYVPELNDVSGNSMLMKGLGSSPYHSVLQPTFHCQKRL

RESSTLFHHYSGNSSNGLTLFDQTPDDTYDKISQSFGFSFLCDSEPDTGSSLPFDVSNGC

HSYINGNFSASKPASGVVKSELPSFQYPETDFGGWGTTSPPQILESVDTFIKSPPQTEVL

QSVCFSPRNSGLLDALLHEAKTLSCGKNHSSDKSSNSSADTPGDAAYSPNRYVSETDWDE

YGDPISPLAQSAASIFSACTPVSGRGSSSDKHPPFEVFTGCNVNVKSETVDQEYTEGEHK

RGTCTQLDFSRPDALLGSGGWLGQGRVTCKNQASMTDTIVSFLGDELGHEYHEQMAAGNS

TSS

>SA07G00070

MKCVFGVISAVNGNNWSSGDGSNGGRRTGSFSTEDYGSLCCGGSSGVFAGGTGGLEGGGM

ERGFARAREGGDDCSAEHAANSHTNGSRTSQLSAEPADPIRACNWSPRDRQNCIKKHYIP

KGGNIRDEDWGNRQTVKRQYRSPNLDHTSLERERRISQSRERERRDMRNPSSSSSTGKGG

GRGGGGGKTTPCCSKVGLKRGPWTAEEDEVLANYIRREGEGRWRTLPKRAGLLRCGKSCR

LRWMNYLRPSVKRGHIAPDEEDLILRLHRLLGNRWSLIAGRIPGRTDNEIKNYWNTHLSK

KLISQGIDPRTHKPLLLNPTSSTPPSPPLTTTNNDNSHHLQSPPSSSKQPNHPLVGNPNV

TPPVHPPQPSDEESPQFLVSLQSGNPHTNGYFDEGGTGFNGNDITIAQNLEISPPIGLRS

SAKINVGLLGLYCEDGEKNDEEDDIGRCCEDEAFSSFFNSLIDENAFTAQHQQLLGGGGG

GGGASLSTTTTDALNISSSAAQAFGLVDPHSENIATSSPSAFFDQQNDHQLRIKDEHKIS

HDKHFC

>SA07G00098

MLSHCASSSSFLSACRVSPPSSAAFLPSPAISSPTSPSPLFTSLKSLNPGRRSRILHRGL

RSPTIGSADTQEEEENRDEYIDVDVSDDDDADESTVDLEALEEEAKSVAREYSLSLSREL

STGDETDFGKGTGKMGILNCPFDIPRPSCMEILGEAKVPDHLLPRVAIVGRPNVGKSALF

NRLVGGGKAIVVDEPGVTRDRLYGRSFWGDYEFMVVDTGGVFTISGSQANVMEELAIKTT

IGMEGIPLASREAAVARMPSMIEKQATVAVEESRVIIFLVDGKAGLSAADVEIAGWLRKN

YSNKRIILAVNKCESPRKGAMQASEFWFSPIPISAITGTGTGELLDLVCSGIEKTEVLDN

LHEKGNYVPALAIVGRPNVGKSSILNALVGEDRTIVSPISGTTRDAIDTEFTGLDGQKYR

LIDTAGIRKRTAVYSAGSTTEALSVNRAFRAIRRSDIVALVIEAMACITEQDYRIAERIE

KEGKGCLIVVNKWDTIPNKNQQTATYYEQDVREKLRLLNWAPIVYSTAVAGDSVEKIIVA

AGTVEQERSRRLSTSTLNQVVQEALAFKSPPRARGGKRGRVYYCTQAAIRPPTFVFFVND

AKLFPETYRRYMEKQLRSDAGFPGTPIRLLWRSRRNMKKDEVMMETRGYVCVISEELDFV

VGRAVMGVGNGCDGGRQLESPKMRKPTDALLGKSSASGNGKNSSNNNKKLRKGLWSPEED

DKLMNYMVSNGAGCWSDVARNAGLQRCGKSCRLRWINYLRPDLKRGAFSPQEEELIIHLH

SLLGNRWSQIAARLPGRTDNEIKNFWNSTIKKRLKNSSSPTPSANTSDVASEQPSDIIPM

RGGGGIMSALQDQALMASFYNLDYSSASSSLTLSRSTTEPIFAVPPLEDLSNSSGYFHSN

ISVPPGVPSVGNASGGEGFYGDERFFIDGGGSFGAVPPLESISFEGNPKTENNILDRNPN

FFSTHNGNNNNKNLNYGGCDGDGGDPLVLESNFGSDGGTCWDLNLAMGEWDLEDLMRDVY

SFPLLDFQID

>SA07G00251

MGRSPCCEKAHTNKGAWTKEEDDRLVAYIRAHGEGCWRSLPKAAGLLRCGKSCRLRWINY

LRPDLKRGNFTEEEDELIIKLHGLLGNKWSLIAGRLPGRTDNEIKNYWNTHIRRKLQNRG

IDPATHRPINELSAGDFTTISFAAAAPAPIKDDPAKIALPVREQCPDLNLELRISLPCQT

QLEPLKTGGNRDWDWDSGSSSGAGAGGGTIGYDFLGLKSGVLDYRSLEMK

>SA07G00478

MEVLFRDSGRIKGPWNLEEDEALQKLVQLHGARNWSLISESIPGRSGKSCRLRWYNQLSP

KVERRSFSREEDEIIIDAHAKFGNKWATISRFLHGRTDNAVKNHWNSTLKRKLSSLIQQG

GSVDTHRFRPEKRSASFGSGVPVPAPCFSPGSPSGSDISYSGLSVMPSSLLFQPAAGTKA

LLQLSKKVEASWKTNDPFTGLSLSIPGTGSQESPSQYLVRDPHQSQPAPPNQYRSKSPSE

SEGRHAMNFGPEFLSVLQEMIKKEVRNYMSGIENNSEACSQTVESIQNALIKRIGMSKSG

>SA07G00630

MGMKNECEDTEMAKDQADSPSFEEASSGGNSGGTAFKKGPWTSAEDSILIDYVGKHGEGN

WNAVQRHSGLSRCGKSCRLRWANHLRPDLKKGAFTPEEERRITELHAKLGNKWARMAAEV

CSVANDKGDSFLMTGWGRIRNPAGYRCFKGRWRERTGKMKGMDRVIERGGEIFEIDRDEE

DDLSQPLVPEKGNRLWRRRMRMPKMDDGGLLRRWSCYPCCRKGVVWEIRQKKFLSNRDRI

SAPQPDKQIRTRAIRYTYLLLPGRTDNEIKNYWNTRIKRRQRSGLPIYPPDMCLQAFSEN

QQSQKISATGDSRLDLFQASSFEIPDLEVKNSELNHGFLSYSPTLFDIPGSRLRPDLGLS

HSYSVMFPTLHPPKRLRESESIFPGNSVASFNQCEKNAYDRIGQPFGFSLYDPNLGADHR

SALGGAFSGSHAALNGNSSSSKPISEAKKSELPSLQYAESQLSSWDVPPSPLPSLESVDT

SVQSSPTEERKSGCVSPWNSGLLDAILYQSRSLKNGNTNSCQKTSSITIGPSEVVNSSSP

EFWASKCDASGDPISPFAHSTASIFSKHTPISGSSVDESLPVETMLGHKVEQGADDMVLL

TQNSERKETADKMDFSRPDALLGYDYWFGLGISQEPLKLAKISASNPKLWILIGIGVAGI

VVLAETTRRRRRRSKFLVTEDFGAFVERLELLPFPQPPPPASRQLLSGLTFAINDIFDVK

DCVTGFGNPDWKRTHEEAAKTAVAVTSLLKNGATCVGKTVMDELAFGMTGENLHYGTPSN

PQMPSHTPGGSSSGSAVAVAAEVVDFSLGTDTIGGVRAPASFCGILGFRPSHGAVSAIGV

IPNSQSLDTIGWFARNPSTLHRVGHVLLQLNPMEPKRARRFIFADDLFQLSKVPKHKTIQ

VVSKVTETLSGYQPPQHMNLCQHIVSNVPSLKSFREQSTKLLNGISALKALSSVMISLQR

YEFKTNHEEWLKSVNPRLGSDVSGHVAEAFNSTYENIKIFYKVRAEMRAALHILLKVPPN

FLDYTFYSMYLSSLSFIISNNAQDDGVLVTPTVSDCPLKINSKKGPSAEFHDKALALLSI

ASMSGCCQVTIPLGKHDGCPIAVSFIAFHGADKFLLDTVLDMFSSLQEQVSIATNAVPLP

DTNGNMDTSELLKEKAFFFELIGKRAGAAIARSASSASYLLSRLCLSFTFRLTSMGLPMG

WSLSQGNAAYKGRQWNKAVNYYSEAIKLNEANTTYYCNRAAAYLELGCFQQAEEDCCKAI

SLDKKNVKAFLRRGTARESLLCYKEAAQGEKFSALSAAFYVMHWIQNEGEGGELAPTPLI

EQKRGMPLIHVWRWRWRKYKNGGLIEVLRKLNDFKHALVLEPQNKVANLAEKRLRKLLS

>SA07G00882

MGAHYEEDPPLQMEGGDGRGLRRGAWTTEEDLLLKSCIQMHGEGKWNLVPHRAGLNRCRK

SCRLRWLNYLRPNINRKKFTWDEIDLMIRLQKLLGNRWSLIAGRLPGRTANDVKNYWNTH

LLKKTANPSSPTKLKKWPNLHNVSSSSNWDTIIPKPRALKPQPRRFKKNLPLCFNLKVPP

STDPTPASTGFGAGNCQAAPPPPENIDISWWESLLFSEETTTQHQQQPLNSSNFVEESAG

TEMLTAEAAEWNCLLEEEQMGWNTDLSFFGFRASEFLCPLNTFMLYM

>SA07G00894

MGRQPCCEKVGLKKGPWTGEEDQKLINFILNNGQCCWRAVPKLAGLLRCGKSCRLRWTNY

LRPDLKRGLLSEIEEQMVIDLHAQLGNRWSKIASHLPGRTDNEIKNHWNTHIKKKLRKMG

IDPLTHKPLPTTSPTDHHPRTLQQEDPIPEKNKEGEGETETSVPSSSIVTATTSNETKGE

GVFDSIGAINSFCTDEVPLIQPHEILPIPTPPPPPPSTSSLTPSSSFSSASSSMVTEKLQ

QLADFDSWLSDFNSSTGNMDFRWDADGFSSWDFLLNDNDIDRNIIIDPLSQYQRLGLDQE

SWKLEFL

>SA07G00902

MGRQPCCEKVGLKKGPWTGEEDKKLINFILNNGQCCWRAVPKLAGLLRCGKSCRLRWTNY

LRPDLKRGLLSEIEEQMVIDLHAQLGNRWSKIASHLPGRTDNEIKNHWNTHIKKKLRKMG

IDPLTHKPLPTTSPTDHHPPALQREDPIPEKNKEGEGETETSVPSFSIVTATTSNETKGE

GVFDSIGAINSFCTDEVPLIQPHEILPIPTPSSSSSSSSSASSMITEELQQITDFDSWLS

DFNCSAVNMDFNWDTDGFNSWDFLIKDDTDRILSLDQESWKFEVL

>SA07G01352

MGWGVMDEGWRKGPWTAEEDRLLIQYVRLHGEGRWNSVSSLAGLKRNGKSCRLRWVNYLR

PDLKRGHITPHEESIILELHARWGNRWSTIARSLPGRTDNEIKNYWRTHFKKKTKGSSNN

SSDSEKTRVRLLRKQQFQQQQQQQQQQQFDGTDIKRIMSLLDEANNRDLSLPHMRHEMTT

TMHTEDQGFSSTCPIFSSEMCVPEAMNEECVLWDGLWNLEDIHAALPMQQQSKQLFPVSH

FQ

>SA07G01407

MVRAPCCDKKGLKRGPWTPAEDQLLITYIQNYGHENWRALPKHAGLLRCGKSCRLRWMNY

LRPDIKRGNFSREEEDTIIELHEMLGNRWSAIAARLPGRTDNEIKNVWHTYIKKRKSQAA

QNQNLTAELSPSARDSSENAMSENSESSSQEQSSGESSAAGATEESPTVPPINDSFWSDS

IVWEPDFPVEALEFLRFPCSPVRTVGPSGCEYSSCSDSGDDMGFWYNLFRAGTLPAELPE

F

>SA07G01736

MEVSQVRGISSRSSSPQKSSNEEESMELRRGPWTLEEDLVLRDYIYLHGEGRWNSLARCA

GLKRTGKSCRLRWLNYLRPDVRRGNISLKEQLQILELHSRWGNRWSKIAQQLPGRTDNEI

KNYWRTRVQKHAKQLKCDVNSKQFKDTMRYLWMPRLVERIQAASSTAGPQLDPAAGHALA

PPPPTQLDGGFANNGYGYGYGTPENCSSTGASSDSFGPQVSPVSELADNYNFPVNSIHPD

TDYYIQGGQQQHHSLSQLNSGDQFSESLISPCGYLNDNTTYGLENFQSVEQSNNQWFDSA

YAATEPDNLWNVDDIWFLQQQFGNASM

>SA07G01817

MVQEEIRKGPWTEQEDLQLVCFVGLFGDRRWDFIAKVSGLKRTGKSCRLRWVNYLHPGLK

REKMTPHEEQLVLELHAKWGNRWSRIARKLPGRTDNEIKNYWRTHMRKKAQERKQAVSPS

SSSSNSCLLSPNYPTADSLPFHETGERSFYDTGGHDTVVSDGKTNEQEEDGEKGYSMDDI

WKEIALPGDEPIKPVDCGPMEKECGFPYPPMASPKWEYCPDSLLRMDEEIEFIDIRAEFV

VSAFSDMSILICSSDLRLNVLFEPESLIWLFRRNALKKKARGFLFSLSPPLPQSTMSQLS

KLYLFAYNSVQAFGWALSLSKILSGFALTKSLSTAYASAGDLICFLQIASFLEVVHGAIG

IVPSGVLLPLMQWGGRTHFLLAIVRRIVEVQELPSVFITFVAWSLSEIVATERGLKVQRR

IINFIGHLSPRDWSALGSFEKMVLRIWRKMWLMYQALAFIKKKNLYADFFAGLPFSYYDF

VRAKTVKTGEARREEEDLKVRLFLQVQARREAGCLNLMNAKVEFGAEDWAHQSNGP

>SA07G01915

MPIHTSSLIIKRHCNRGDFLLAAKKLQDRSKLKIQNSVLGKERERERGRGRGGEMGRAPC

CDKANVKKGPWSPEEDLKLKDYIHKYGTGGNWISLPQKAGLRRCGKSCRLRWLNYLRPNI

KHGEFSDEEDRIICTLFASIGSRWSIMAAHLPGRTDNDIKNYWNTKLKKKLLAMQLLPPS

PPPPLLSSPSSSSFHHLETAQHTSSPSPSSLHRDYNPMLMMSPYDYHHHTPATPFLQNHG

HYYQEDAVKENYNGSREIKQEEGIGVYMPSNDTNIGENSNEALFCNSWFDHNPIMMNDGC

FGQTHQLGLLQEDVLHAEMEEPLGIGTCNSGFFFSGGESKATASTSTSTGYQSEEVVMYH

>SA08G00096

MGRRPCCSKEGVTRGPWTVLEDNILAGYIRVHGEGKWRSLPIRAGLNRCGKSCRLRWLNY

LRPDIKRGNISQEEEDLIIRLHNLLGNRWSLIAGRLPGRTDNEIKNYWNTHLAKKTTLSS

SSSAPSGNPKITISPTPTPTPGEKMKKKPNDNNDEPTIISPTQLQKPIRTKPTKRTTGVL

FPPLLPHLPLDDHCITSPSMIAAGKEPIRATTNVPSSAGLLTPDHDGSRSIGGNKDNEDD

EHARLLDQPIFDEDWVADFFNEMEENVTGFSIAANDA

>SA08G00440

MVIIFIKQSAEPVKHLPGEGLLLTEHQGTGQNRKHEAQGLCGSHTWCAAVLIEVPFKESS

TAHYNPLVDLETKYTARFRKMSADFEWGVSKEEQRRKKGPWTHEEDRLLTTYVSLHGEGK

WTSVAKFSGLNRSGKSCRLRWVNYLRPGLKRGQITPIEEAIIMELHALWGNKWSTIARCL

PGRTDNEIKNYWRTHFKQVRPPQKEGKEEGMRQKSSQQQQPQGNQGDGAEMVSPELEISD

MRMDSEPQAPPSDVGFTYSTIEDQYYLPSAMAQDTAAWWDSSEEDELWSGLWNLDDQLH

>SA08G00533

MGRPPCGDKVGVKKGPWTPEEDIILVSYIQQHDPSNWRTVPTNTGLQRCSKSCRLRWTNY

LRPGIKRGNFTDHEEKMIIHLQALLGNRWAAISSYLPQRTDNDIKNYWNTHLKKKLRKES

SIQQQPIARGQWEKRLQTDIHMAKQALSEALSIDVPHDNAPSDSNPSNGGDYSYTKPVQP

CFYASSTENISRLLGNWTKSSPNIVKDNPDAHAGTYHPNSGFSADESKPLAMLEKWLFEE

GSVQVKGEGNCEFSSNGNAHEVGSSFWGYEWGSVTAS

>SA08G00634

MDKKACNSSQDAEVRKGPWTMEEDLILINYIANHGEGVWNSLAKSAGLKRTGKSCRLRWL

NYLRPDVRRGNITPEEQLLIMELHAKWGNRWSKIAKHLPGRTDNEIKNYWRTRIQKHIKQ

AENFTGESCGELNDNFQGSTSHHQMESVAIDHHHHGLENYSPPSFPSNEDFQRSMNNHPH

QALTGPSSSSSVSVPNLPGESNDNLWGMEDLWSMQLLNGE

>SA08G00642

MEFGTNSRDDLSLIPPPSLNHNFLRMGNNEDGPTLVENVIPHRELATETRFRFQGSQSHH

HRRHLDNGLDHFSIDGFSSTNPLFGIPPTPPCLDKFLFGGGDKASSNNGTAFPHEFQRFP

TPYSSHVMMNMIMSPPPPPPPPHLPPVLRLNNNYQEFGCLRVGDEVSCITGESHGNYSER

NINYDLGNKEGRSDDLMVMMMMMKKKKGKLQAKRTKFKGPMNSSNVIKGQWTPEEDRILV

QLVEQYGVKKWSHIAQVLNGRVGKQCRERWHNHLRPDIRKDIWTEEEDKILVEAHKDMGN

KWAEIAKKLPGRTENTIKNHWNATKRRQFSKRKSRKHPLNSLLQNYIKSVTTTATSSSSS

PHRTMDPTHPPNNASSASDISNDNPNLQESLGFITPNRSAIQSPATDAPNYRGLDEIMDL

SFGQNVAFAERFGLGPGLEDVPHGSEVESSICNTTATTHVGSLDFKLPSHEMDNQMQSHL

VKREMDLMEMLWQKRNF

>SA08G01393

MGRPPCCDKANVKRGPWTAEEDAKILAYVATHGTGNWTLVPKKAGLNRCGKSCRLRWTNY

LRPDLNHSSFTSQEEDLILKFHKAIGSRWSLIAKQLPGRTDNDVKNYWNTKLRKKLTDLR

IDPVTHKPFSQVISEFGVIGGAAPPNPASTVPVGPPMVPESAYYSSRAAAINPNFPLSAF

RDGVVTPPVGTDFRFGSSDPLRDEKFQSDHANGNENGGFGALGYEAGSSSCSRSLMVGSS

FVEEILDRDSEMQREFPCAFMDESFYC

>SA08G01401

MGRPPCCEKVGVKKGPWTPEEDIILVSYIQLHGPGNWKAVPTNTGLLRCSKSCRLRWTNY

LRPGIKRGNFTGHEEKVIIHLQALLGNRWAAIASYLPQRTDNDIKNYWNTHLKKKLKNKI

QTGPNNELRGDGSSNQDPIPRGQWEKRLQTDIHMAKQALCEALSLGKSDTNLASELKPSG

GYYSFTMPSQPIAYASSTENISRLLENWMKNSPKTNPSVTQHSSAKSARKGTECTAWEQN

KESGDQLSVAFGSLFGFEPFDSSGSEFSPDESVLQDGSKPRGQVPLLMLENWLFEEGSVQ

GKGEGLAEISLDGDVHDLF

>SA08G01896

MSRRGGGAALQKDAPWRASSSVKPIPKIHHSPVLRLAHTPFSDYALSLMKLPDPIGSGLA

TEAIVEAAGPDCIIPGQITPVKLLGLKVVTIGYGPLPVSLSEAVFVSIIVLKTVVAGNSC

VFRVEFRLFESASNERGMVLRKGSENEEHCLLSNCMYVNNMASQAAMREVKWRKGPWLKQ

EDCRLIAYVTILGERRWDWIAKISGLKRNGKSCRLRWLNYLRPGLKHAPMSTEEEQTILQ

LHERWGNKIAQKLPGRTDNEIKNYWRSCLRKSAQVQKEHKHGVAKSEKQNILMQEGNVGN

TVNDFDVAGLSSSTFTNSAYESHVLDWTVWLSIAEHHKNGTDFCFCCPSKWCSEDDGSIL

RDWSASMWENSRT

>SA08G01905

MGRAPCCDKANVKRGPWSPEEDKKLKEYIEKFGTGGNWIALPHKAGSTSLLEEGLKRCGK

SCRLRWLNYLRPNIKHGDFSDDEDRIICSLFASIGSRWSIIAGQLPGRTDNDIKNYWNTK

LKKKLLKVPFSSTSSSSSSSTSSHTTPSSSSSMYQCSSSSNPTYNFYNYNSSGCYYEQHP

ISAISCLSDNTTTVSWTPSHYMQSQHLVEDTTTTSSSGGDGGGVSINDQDMGLPSLFLCN

ITGEDGWRNGDEGLNMNWSWGEEVPLMDYGLEEIKQLISTTTTTNTCNNILSFNQE

>SA09G00124

MGRAPCCDKANVKKGPWSPEEDSKLKEYIEKNGTGGNWIALPHKAGLRRCGKSCRLRWLN

YLRPNLKHGEFSDDEDRIICTLFASIGSRWSIIAAQLPGRTDNDIKNYWNTKLKKKLLMG

TIPSSSSPIISKPHHHPYSPQLSSPSPSSTSSSLHAGMPSSTSSSSSSSSSSSMYQCSPS

TGYYYYEQQQQQQQQQPISALSSFLNNTSISCTASHHMLSSFLNNNSVSCTASHQQVRDS

TSNLFMLGATTTATTSSSSHQDMGFQGYYCNNGEEEGQKFMVSSNEGLWGGGTEMPLMDY

GLEEIKQLINTTNNSNNVCNGISFDQEIKTEMEGVVYL

>SA09G00284

MGRQPCCEKIGLKKGTWTVEEDHKLMSFILSNGIHCWRMVPKLAGLLRCGKSCRLRWINY

LRPDLKRGTLSESEEDQIIQLHARLGNKWSKIAAHFPGRTDNEIKNHWNTRIKKRLKLLG

LDPVTHMPIHQNHTTDEDNTVDTIPHPASSKQENETSDANSGVDHPTRAEEKQREVDLTF

GESTVLLDDHDETLGAFDVGLWLNQENSIPKVWSPPVSVEDSLNPSAGESSSSFPEESLQ

QWINGEDSLLSWDGFNQLQEELFFMDHSNEHT

>SA09G00324

MEFATNLSSDSNSLYMENGGTGVSMETRFPFQGSPPNPFFGIPTPPYCLGTFGGNGGMGF

HEFQRFGPPHGNSQMMNSVKGPHPTLSVNNSSHETEAFCITGENNASNIDYGSDDRVLMM

EAYVQGIRKEKGCKMTANVIKGQWSPEEDRMLVQLVEQYGVKKWSHIAQLLNGRVGKQCR

ERWHNHLRPDIRKDLWTEEEDRTLVEAHIVFGNKWSEIAKRLPGRTENNIKNHWNATKRR

QFSKRNSRKHSMNSLLQNYIKSVTPPSTLSSTPTDKSMDLILHQKNASSSSGISNGSKST

DFGIGNRPAESEAQPNHHDFDEIMDLFSSKFMLAERFGFRSAIEDMSCGSIMEHKSNYNT

HEVSLDFNMPFS

>SA09G00373

MGRPACCDVVGVKKGPWTPEEDIMLVSYIQQHGTGNWRAVPTNTGLLRCSKSCRLRWTNY

LRPGIKRGNFTEHEEKMIIHLQALLGNRWAAIASYLPQRTDNDIKNYWNTHLKKKLRKVQ

AGSSDGLKGSTQQHPIPRGQWEKRLQTDIHLAKQALCEALSFDKSDDFGSSSDLNPSKND

FYCHTKPTPPSMYASSSENIARLLENWTNDSPKIAKTNSSASQHSMNDAPAGTDSSSSGK

KNGGDQLSMKFESLFGFESFDSSNSDFSQSLSPEASLLQDESEPADPSAHVPLSLLEEWL

FDEASIQGKGDGGLAEISLDGSVHELF

>SA09G00502

MLYKAVQQFQGKNWKKIAECFKDRSDVQCLHRWQKVLNPELIKGPWSKEEDERIVELVNK

YGPKKWSTIAQHLPGRIGKQCRERWHNHLNPGINKEAWSQEEELTLIRAHQIYGNKWAEL

TKFFPGRSDNAIKNHWNSSVKKKLDSYLASGLLAQFQGPSYVGQQTSSTPSSSLRIQQSS

GEESVPKDGAEAEEISECSQGSGAVGCSQSGSEKANGVLHVREECQTMEESCQGKEQNSN

TALCSGKYYASIEEINYCVPDIQCKQGGSPKLFAQSISHHDGTSMSRLCQYNLHELPNSS

LPTHFIDPHEKHEVIPGVLQNSLGLSSSTSVGIPIMGSEKPEGVLTPKGNTSRIGFAEAG

PGACFSSESLIKCSNIVDLIDCTDSVPYESSNFQKPETARTSVSQLFYPLKWSDMSGSSC

CESFLSSSSILPVDDDTILNSAEPNQLNDLSVGNLKQEIVGKSLDGFLCPNGSADSTFND

GTDAAGLQDLVDSSSKLVHEDIFQLVSSDVMDGHCIDEKPAICAEQGDAGGLCYEPPRFP

SVDIPFFSCDLIQSGSEMQYSPLGIRQLMTSSVNCMTPFRLWDSPSKDDGPDAMLKSAAK

TYTCTPSILKKRQRDLLSPLSEKRFDKKLASNFSQEFSTSSLARDFSRLDVMFKETESPK

ASPCSPSYNENKKLEPHSEDKENICPEMGKEEKRDGTGKVNADADAQVVEQSYGAFIECN

LNDLLFFSPVQVGLKPERVLGSSAASSRRHGKESQVASNQITFPQSSARNQCLSDISSPT

VYKKKHDNHPTAVPPRNYAPSTDPDVTAENKRNIAGIENFNMFGETPFKRSLESPSAWKS

PWFFNSFFPRVNGELSIEDFGYFMSPGDRSYDAIGLMKQLNEHTAATFANAQAILGNETP

ETILKERLSNHQNTEQENNHAANGDLKNSPPFSSNILGERRILDFSDCDSPAKETENGKG

SAAISFSSRQFISRQWPFTSFTQEHFNSSVEKVQRGGKRGSHCRYLSESAVELGGGGREG

SSFQTLRLPEDFFSLTKTQQFRRTVSLSESKRSSLLSDEDRAMDPPTSWDSLRKQARRLE

AQLDEQMHLYRRLVSARVGDVKENDVESGIDTSLKQLQQVNSHMQAWVSSGGSEIFSHTL

TRHQEILHELFQEFYRLRSNLKAKKEHASLLENFKEFDRARADLEDGGGSDGQALLKEHA

SISRSSGQEEIEEVEGGGGDQVEEAAGGLNREEEVSEEEIWQRGPLEEEMDTVISQAQAT

LGSLVFQRSTFGGIDSKLSNISSHLPTENLLLVEPYWHDLLSVKLSILNAF

>SA09G00690

MRSPCCDESGLKKGPWTPEEDQKLVDYISAQGGHGSWKSLPKLAGLNRCGKSCRLRWTNY

LKPDIKRGNFSEGEESIIINLHSSLGNKWSKIATYLPGRTDNEIKNYWNTHLRKKLLKMG

IDPRTHRPRADLNFLMNLSQFLSPSIGLGLTSSVGRDMSLLRLLQADATQQAKTQYLQNL

IQAISASSVPYYNNSLDQFQGLIGNNGSSFQSTIDPLQALLGLPSPLLGLPLPAGDADAI

SKCNPQEEKLSENNASDGCSLWDSNWYNGLQDEGNTLPELVSASEFENSQLMVNEMGSIT

TTDKASFSSNVFGDCNNWKKVMDDEAGGSFWEDIIH

>SA09G00869

MAPKKVVESGKEALNKGTWTEEEDQKLAEYIEVHGPKKWKTVATKAGLNRCGKSCRLRWL

NYLRPNIKRGNISDEEEDLILRLHKLLGNRWSLIARRLPGRTDNEIKNHWNSHLSKKLMD

QKERTLRTSPAQEARSPKISNMKDEGTIEVSNSDIGFDVDKFFDFSTEGTSGLEWVDKFL

EQEEDPWIMRRKGECDAVDSNCSSQGIS

>SA09G01856

MGRAPCCDKNGLKKGPWTPEEDQKLIDYIQKHGYGNWRTLPKNAGLQRCGKSCRLRWTNY

LRPDIKRGRFSFEEEETIIQLHSILGNKWSAIAARLPGRTDNEIKNYWNTHIRKRLLRMG

IDPVTHSPRLDLLDLSSILNSPLYDSSHLNVSRLLGIQPMVNPELLRLATHLLSTQRESS

HNFLAQNAQGNQVQTQVQPDQITSSSVQEIPPCGGSFGIQAEAPQPNVDQYPSSFTHFGP

ESSLNEWKTPCHDLIDQDYASLTSYANYGSTDQGTVDPLPDYNSIFSNGYHGGGNPNFGF

GPVLSPSVSSPTRLNSNSNCMNSSTEDERESYCSETLKFEIPESSKPSTEPQIPRTNKVA

ERLSFEGHTMNAQDDVGAHGSAPSSDLPIPLVLLNLLPSIDPVLLCGLNLRRRGLGLGSF

VLYFGFCDLLLGGVYQYILRFEIGSEKWNIRRVASNVVAQRRRSICSESRRTYVRLVAPE

QQFPKYIFCRHLPLSGSARKFPKTNYSRNLSFVSGSNTTAHHAQVAWRRISQINSNNDCV

HSRISKIAQAFSLASTRSHLVAPGIFGFICGELALSNRAFAETEHFSPGNTIYMRAQDGH

AFAISFFLSLIEGLVMLLRALYLVFLFSPSIVIAPFADSFGLQFRKSWLQLVHHTLERAG

PAFIKWGQWAATRPDLFPRDLCSQLTELHSKAPQHSFAYTKKTVERAFGRKISEIFEDFE

ELPIASGSIAQVHRATLKFKYPRRKGKPAVVAVKVRHPGVGESIKRDFAIINTVAKISSC

IPTLKWLRLDESVQQFAVFMMSQVDLAREAAHLSRFIYNFRSWKNVSFPKPLYPLVHPAV

LVETFEQGESVSYYLDELQGHDRLKTSLAHIGTHALLKMLLVDNFIHADLHPGNILVREA

QSKSSRKQLFKSKPHVIFIDVGMTAELSKSDRVNLLEFFKAVARRDGRTAAECTLRLSKQ

QNCPNPKDFIQEVETSFNFWGTPEGDAVHPADCMHHLLEQVRRHRVNVDGNVCTVLVTTL

VLEGWQRKLDPGYDVMHTLQKLLLKSDWAQSLSYTIEGLMAP

>SA10G00158

MGPHSCCNKQKVKRGLWSPEEDEKLINYISTYGHGCWSSVPKHAGLQRCGKSCRLRWINY

LRPDLKRGSFSPQEAALIIELHTILGNRWAQIAKYLPGRTDNEVKNFWNSSIKKKLLSSH

SISTTASLSHHHQLPINPNSATSTENYSPQDPSLFSNDFIPNPNNNNNNIHDQQSHLLLP

TPPIPLLMLHEGFDELDSAVCPMILPMAPMPEPADWYSQNPSLTPMDYYHQLLQYPHNDP

VFHTLEGGPHGATEPGMTAAAAALPHDNMGMPKLCEIGDVKEYCGSVPSPSTDVVYSKFS

CFCNSESNYGYVDRSGEPVDPVEHPEGCIPPSVSMTSASACSQFVNPSYDNSLPADPSCW

EF

>SA10G00182

MAYQNFVGGSTGKNAFYGSFCEIFRANDGHILANQEITGALMNLRTCSSIADTSRTIYDV

FIRYHVLNQTPQRQLAKSPQNPQRKAQSFCRSSYPDRVAVAAPEFCPLKIAGFPHWEKEK

DPPHFPVQWCEEAAVVPDEEVEERKDAAAMEAVIEAAIEEGAAFEEGEEMKVVAGKEEVA

TEVEAMEEVAEAEAEVETLSAFDFEEEIAVRIWLSEISDAPEESAQRVKWVTGKTIDLMV

PERMWVWGAQGTFSPFQRDVTDSRNRDEEEDDEESSTVRQIWVEIGIALTEQNSEKPLMG

NSRDRKEASRGAWTAEEDQKLAETVAVHGARKWKTVASKAGLSRCGKSCRLRWLNYLRPN

IKRGNISDQEEDLILRLHKLLGNRWSLIAGRLPGRTDNEIKNYWNSHLSKKLKQSQKQVM

ASEESREENAVKRSGTEESKSSTHGADPSSFDSCNEGPLSLEWVSQFLELDEWFV

>SA10G01160

MGSKSMCNSDEEGELRRGPWTLEEDNLLLKYISCQGEGRWNLLAKCAGLKRTGKSCRLRW

LNYLKPDVKRGNLTPQEQLLILELHSQWGNKWSKIAQHLPGRTDNEIKNYWRTRVQKQAR

QLKIDSNSKSFLDAVRYFWAPNMHQTNDQVVSPPHFPSSSSTMNSTMPSPITNMPSSPSP

SAVPGINHADNYASLISPTSMSSAHLLQNSQFPEVLEHPINPYSFDNSFNDHAIPNGNFS

VVDNCSYDMGDYNPHPTSAMGIYDISMSDYPIAAGGDWASGDMEQTVPIWNMDELWQFEP

RIDDH

>SA10G01208

MGRSPCCEPAGLKRGAWTTEEDQKLLAYIQLHGHGCWRSLPQKAGLRRCGKSCRLRWTNY

LRPDIKRGRFNSQEDRTIIQLHALLGNRWSAIATHLPYRTDNEIKNYWNTHLKKRLTQLG

IDPATHKRECFASTISHMAQWETARLEAEARLVRESKRRRIQSSPPKPLRRMAAAATAPC

LDVLKASQIVWSRPTSGAAAAAEARNSGSGFVGRCNPDRPKSVPICSENSFKANTYSGGV

DGEELWKLEGFNITEDGNLVEGFVNLLGDQSFSGGRYSAIDGDGAQEGNREYWSDLLSSI

NCGSPPDLAVI

>SA10G01225

MGRPPCCDKMGVKKGPWTPEEDITLVSYIQEHGPGNWRSVPTKTGLLRCSKSCRLRWTNY

LRPGIKRGNFTDQEEKLIIHLQALLGNRWAAIASYLPQRTDNDIKNYWNTHLKKKLHKLQ

SSSPEGSCYNQGGLLSSSSSSSVSTPQNPKTNSSNGQWERKLQTDIHMAKQALREALSMD

QPSCDSRDLSSGPCVQTPAMYASSTENIARLLQGWVKKSPDMVTLNSSCSQQRSPDALSS

GDSTQQYDDSPLGTPENGVDVKTEAVADRSASQLSLLEKWLFDDNVGCVESPGQEALMDM

PLEDTAQLFY

>SA10G01270

MVTPPCCDKLNSKRALWTAEEDAKILAYVSKHGTGNWTTVPRKTGLKRCGKSCRLRWNNY

LRPDLKHDVFTPQEEELIIRLHSAIGSRWAIIAQQLPGRTDNDVKNYWNTKLRKKLTEMG

IDPVTHKPFSQILADYGNIGGFPKTAGPRIGPLTRDLKNAFRLKTERQSPAVPKNPHLAT

QKVIPPKFEPSGDHSLDLLTELQSITLVKEASTCTEQPSLFDQMKPSSSSSSSTCSTTAQ

AKLGAVGFSWEDFLLEDAFLPEQGNVGEFVPNAVGVHSNKEKSIVGEENENCGGVEYNNG

VQVSSSSSSSFVEAILDQDIEMFTEFPCIFEDPFYY

>SA10G01325

MEETFVPFRGIKNDLKGRLLCYKQDWTGGFHAGIRILAPTTYIFFASAIPVISFGEQLER

NTDGTLTAVQTLASTALCGIIHSIIGGQPLLILGVAEPTVLMYTFMFDFAKDRKDLGPKL

FLAWTGWVCVWTALLLFLLAILGACSIINRFTRVAGELFGLLIAMLFMQQAIRGVVEEFR

KPHNENPDQSALLSYWRFGNGMFALVLSFGLLLTSLGSRKARSWRYGTGWLRGFIADYGV

PLMVLVWTAISYIPVNDVPKGIPRRLFSPNPWSPGAYSNWTVIKEMLDVPPLYVVGAIIP

ATMIAVLYYFDHSVASQLAQQKEFNLKKPASYHYDLLLLGFLLLRNKLVSAAQKSMRKNA

NLGQLYASMQEAYKEMQTPLVYQTPPTLGLKELKESTIQLASSTGYTDAPVDETVFDVDK

DVDDLLPIEVKEQRLSNLLQALMVAGCVAAMPLLKRIPTSVLWGYFAFMAIESLPGNQFW

ERILLLFTAPSRRYKVLEENYATFIETVPFRSIATFTLFQTVYLLLCFGLTWIPIAGVLF

PLLIMLLVPVRQYLLPKFFKGAHLQDLDAAEYEEGPAIAYNMSFEDQAHPRTPHIDDGEI

LDDIVTRSRGEVRRMNSPKVTSSSPSPVDAMKPTYSPHLCQRSLSPRTKELTGKGLVPQS

APSPGPSPLGQSSHGPSQGPKESFEVQSTFEAFLSLVSDQDTSSQQPLFLCDNNYPKLED

GLSLQDFQNLDQLHIPAYGSSSNSPDFGTNIDHFDHALTAYASAATTNNDKNANNILNPY

QSKPFSEQAALDYPYGTFHAGGGGGGGGGGEGEFFRQPLQAVGIDGIGSGHPSNFPYYNC

HGEMVKPVNFVIQDEVSCTSTAVENNNNNDRNGGGGYYKSVGMNNNNNNSGRTRAAASAL

TKRICKGRRKSTVVKGQWTVEEDRVLVQLVEEYGVRKWSFIAQMLKGRIGKQCRERWHNH

LRPNIKKDTWSEEEDKVLIRAHEEIGNKWAEIAKRLPGRTENNIKNHWNATKRRQFSRRK

CRSKFPRQSSLLQNYIKSLNLNSNSSTTTTTTLTPHLPKNRKVADPSSVANNTASADYAM

VPAPSNTMLSTSHQNGADRLVADFDFNDVPDLPFDASIMFEERCGGIDSLFDEENVEMNE

MMMMPLDDDHMDSLMQCEVKKELDLVEMISQVNQ

>SA10G01351

MGRHSCCLKQKLRKGLWSPEEDEKLYNYITRFGVGCWSSVPKLAGLQRCGKSCRLRWINY

LRPDLKRGMFSQQEEDLIMSLHQVVGNRWAQIAAQLPGRTDNEIKNFWNSSLKKKLMKQG

IDPNTHKPLNESEVKNENSVEKPPLHLARCKNISQPAFAPTQLEPTFLVSTSTSGEDFMN

KPEFNPFSLFDYEPAIDSYTPSFDTQNHQNVRPTVDQYHLEANSNTAGFTLMPSLSNFDH

GSLVDSEISDNSTSRISCSNSSNIINMVGNGGFSWDAEKKFESLLQCQLNGVKPEELKPS

SWEEQGQNLHSYNSDNFSSYFFTSLSEDLTVPNFDAFQHI

>SA04G01993

MGRKPCCVKGEVKKGPWSAEEDEKLTNFILNNGGHRCWRALPILAGLNRCGKSCRLRWTN

YLRPDIKLGCFTDTEEQLVIDLHALHGNSWSKIAAMLPGRTDNEIKNRWNIDIKKKLIKM

GIDPSTHKPLQENEETGGQIEALSRLGASPEINVDQSPKSPENFKTEEAQSPENSKNDET

QPPEPRGATAATDEDYQKLLMSYWAQSKFLGDDFMPPAGLEYSNEFGPSSSGNDFGFHPC

KSSSPISPSPTPAKPSLPIPPPPPVAVSHISYAVPNFFIWFPSTNCRSLNCCLQLVQKSN

MGINIE

>SA04G01984

MGKKQCCVKGEVKTGTWTPEEDGKLVNFILSSGGNRHSSWQRVPKLAGLSRCGKSCRHRW

NNYLRPNIKHGPLSDVEKRRIIDLHAFHGNSWSKIASMLPGRTGNEIKNQWKTNIQKNLM

AMEIDPYTHQKLHENKDRSEQTPSHLLASSENDAHRLSAESWAYIGDRECNDRGQLPEQN

NAFTIDDSDLCALMTKIELEMFLDDTSCMFMQAREEYNNYFGVSSSSSPSGRFCEVHYTG

MV

>SA04G01982

MGKKQCSVNGEVKSETWTAEEDGKLVNFILSSGRSRYSSWQRVPKLAGLSRCGKSCRDRW

NNYLRPDIKHGPLSDVEKQRIIDLHALHGNSWSKIASMMPGRTGNEIKRQWRTRIQKDLS

STRTDPHTHQKMHENKDSVEQTPSTLCDSSENDAHLSLAKSRAYNGSCESNDRGQLLKPN

NAFNVDDNDLCVLMTKIELEMFLDDTSCRFIQAREEYDNYFEMSSCSTSSGEIL

>SA04G01985

MGRKACFVKGEMKRCVWTAEEDERLSNFILNNGSHHSWRILPRLAGLNRTGSSCRLRWAN

YLRPDIKHGFLTDAEEQLVIDLHALHGNRWSKIAAMLPGRTNNEIKRRWRTNIKKKLIKM

GIDPVTHKPLHEHEESSQRETPFVSDHPPKINVCQPDKSPENSRLPEPCSATTVDADFID

QLMSYWVQSKLLDNDFMPSAAGEDFNDEFGSPSSLNDDFGGEEDSGFDSFNDLWTY

>SA02G00633

MGAGHLDRIKAPWSAEEDEVLQRLVFRLGTANWSLISRSIPGRSGMSCRRRWCSQIPPGAEHRPFTAAEDEIIAQSHAVHGKKWATISRLLAARTGHSIRDRWESTLKWKSSSRIGGGGSAGKSRFGPASSDSDSPASQRIDASSETEDPLTRLCLSLPGTESDESSSN

>SA04G01975

MGKKECSVKGGVKTGTWTAEEDGKLVNFILSSGGHSSWQRVPKLAGLSRCGKSCRYRWNN

YLRPDINHGPLSDIERQRIIDLHALHGNSWSKIASMMPGRTGNEIKNQWKTHIKKDLRAI

RVDLLIHQKMHENKYRAEQTPSPFCESSENDAHSSSALSPTYNGDCESNDRGQLLEQNNA

FTIDDSDLCVLMTKVELEIFLDDTYCTFIQAREEYNNYFGLSSCSVSSGETL

>SA04G01963

MGKRQCCVKGEVKNGTWTAEEDQKLVNFILARGGNPRFSWLALPKQAGLSRCGKSCRFRW

INYLRPDIKHGPLSDAEKQQVIELHAFYGNRWSKIASTLPGRTGNEIKNQWRTRIKKELM

ATRIDPLTHQKLHENKDRAEQNPSPLLDSLKNDARWPSAKLPAYNGGCESNDQDQLLRQN

NVFVVDDSDLCLLLTKIELEMFLDDTFYMFMQAREEYHNYFGISSSSSSFEKIL

>SA08G00227

MSTASLSKETVQKVLRQVEFYFGDSNLPRDNFLKKSISESEDGNDVSEETVQAVAEVLRQ

SSSLKISEDGKKIGRATELSKPEEVIEQLDARTIAASPLAYDTNIEDAEAFFSQYAKVNS

VRLPRHVADKKLFCGTALIEFAAEEDAEKVLKQSLVYAGVDLELRQKKDFDAKRAIEAEE

AEKLRSITGSNHGKSSNAGVNNYPKGLIVAFTLKRMSAGGSQEQSSVPEVANDNEDGCKT

NGKLDSTENVSAEDVSGNENNSKNEENHEENNEGSVEKGDEKTSSESVGNENEDGSVQKD

EEKVTTEGAFDAAACKDNKDIVMREDLKEVFKKFGTVKFVDFKIGSESGFIRFEGPEAAQ

KARAAAVLAEEGGLIVKNYVATLEPVTGDAESEYWSLLRGNQEKHRDNKGGRGRGGKYNR

GGRQFHGKQGRSRENDSSSDRPDKAQKVGEELEKVGIVFFVASCIAVKVVPPLRCVLIQS

VWSEPRRKSKKLTNSRSLANVNDTGTMESDYTTIIQDGFSDSLQKTRPSHGRTSGPTRRS

TKGQWTAEEDEVLYKAVQQFKGKNWRKIAECFKDRTDVQCLHRWQKVLNPELVKGFWSKE

EDERIIELVNKFGPKKWSTIAQHLPGRIGKQCRERTDNAIKNHWNSSVKKKLDSYSASGL

LGQIEGPPYVGQQTNSMYSSSLIMQQSSGEAVPKAEIEAEGVLECCQGAGVGCSQSGSDE

ANGIEHKGLKYQMNEESCRGKEQSSGPTSCSEQYFTSMEEIEFSVSDIPCELDGSPMFLA

QSSSHGATSSANRHRQYNLHSFPDSSSLELEHDSNLPTEFIDPHETRGNVDSLGLIPSAS

MGHSIIYSEKTQEPFMPKEGCCRVNLPEAGPGACFSSESLIKRSDSVDLIDLTDSLPVGV

SISQPYHPMKPSEMSGTSSCQSFLSGSTLQVDDVAYDAELNQLNNMLVETHHRQQDFAED

SSKLVNLATSGLGSLDVMQGCVDEKPVFCAAEKGNSGGLCYEPPRYPSVDIPFLSCDLTQ

SGSDMQQEYSPLGIRRLMMPSVNCSTPFRLWDSPSGNSRADAVLRSAAKTFTSTPSILKK

RQRDLLSSSPEKRNDKKLASDFSWEFPASSLARDLSLSDVMFEEGENFKLYACSPSFSEK

RALEAYDEDKENICPGVGEEEKIDSTVKAEANAVEESSGVLEHNLNDLVFFSPVRVGFKS

DRVLGSSAETSRCHFGKESEEALHQSTYSQNSARNLCLPDASSPTVYKKKHDSHFSVVPH

TQFAPSSTSLDTITENNRNSSGVENFNIFGETPYKRGIESPSAWKSPWVFNTSFPKANGE

LTIEEYGYFMSPVDKSYDAIGLMKQLSEQTAATFANAQAVLGNETPETILKQKLSNLWNS

NEGKDQVSYSDPESSSHLASNVLTERRTLDFSDCGTPGKGTENGKLGAAISFSSPSSYLL

KGCSNEKPFSSV
